# Supplementary material for: Nucleoporin TPR is an integral component of the TREX-2 mRNA export pathway
Source: Nat Commun. 2020 Sep 11;11:4577. doi: 10.1038/s41467-020-18266-2 (PMC7486939; doi:10.1038/s41467-020-18266-2)
Supplement: Supplementary file 1 — Supplementary Information [file 41467_2020_18266_MOESM1_ESM.pdf]

Supplementary Information

Nucleoporin TPR is an integral component of the TREX-2 mRNA export pathway

Aksenova *et al.*

Supplementary Figures 1-11

Supplementary Table 1

Supplementary Methods

Supplementary Reference

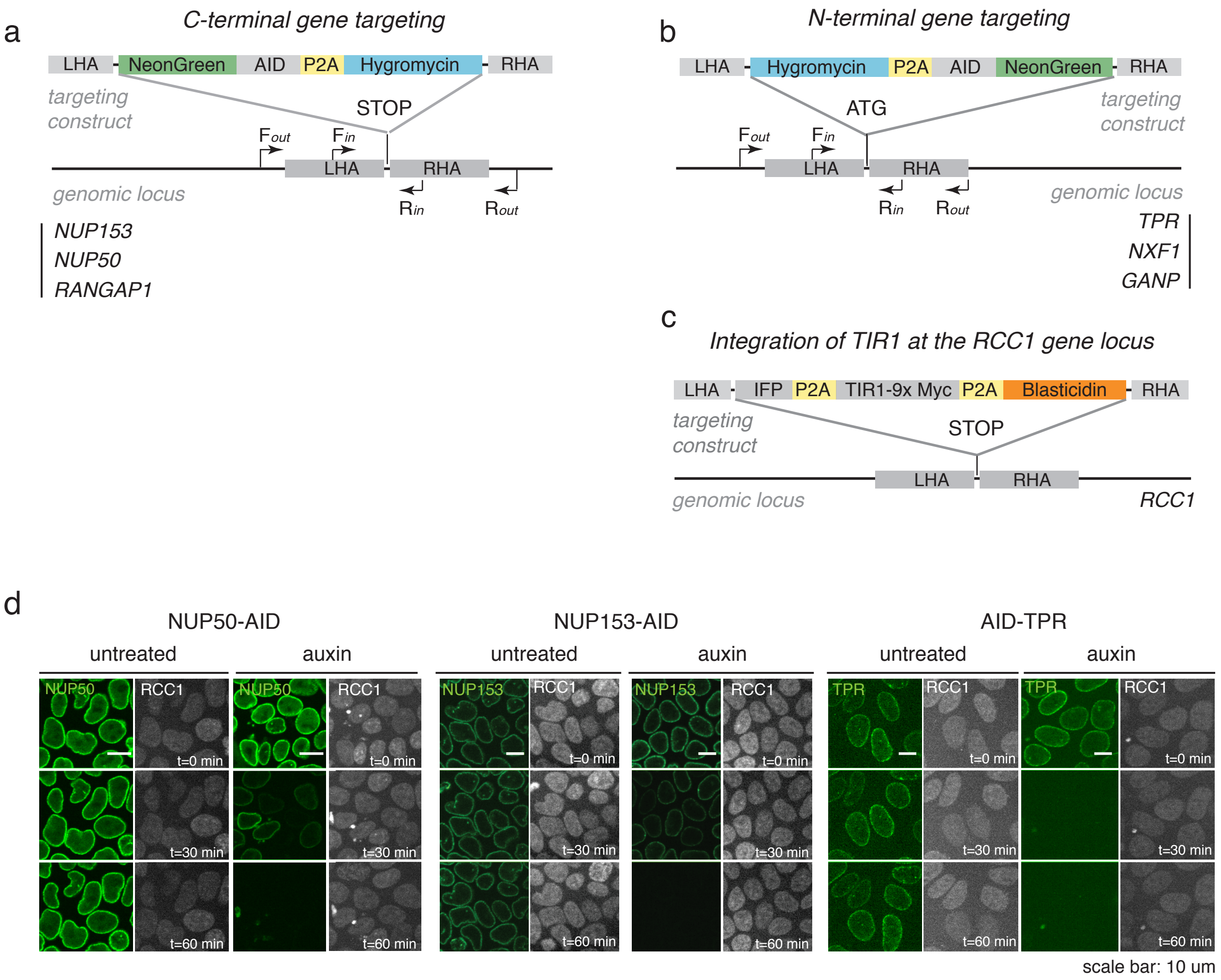

Supplementary Figure 1. Gene targeting strategy. a-b, A scheme of endogenous targeting, position of homologous arms, and primer sets for genotyping of C- (a) and N- (b) terminally targeted genes. F*out*-R*out* primer set anneals outside of the homologous arms, F*in*-R*in* primer set anneals inside of the homologous arms. **c**, A scheme of TIR1 genomic integration into the RCC1 gene locus. **d**, Live imaging of NUP50, NUP153 and TPR degradation. Chromatin is visualized by RCC1-iRFP fluorescent protein. Scale bar: 10 μm. The green fluorescence intensity at the nuclear envelope corresponds to tagged nucleoporins in the absence or presence of auxin. The median degradation times for NUP50, NUP153 and TPR were 60, 40, and 20 min, respectively. LHA – left homology arm; RHA – right homology arm, IFP – infrared fluorescent protein.

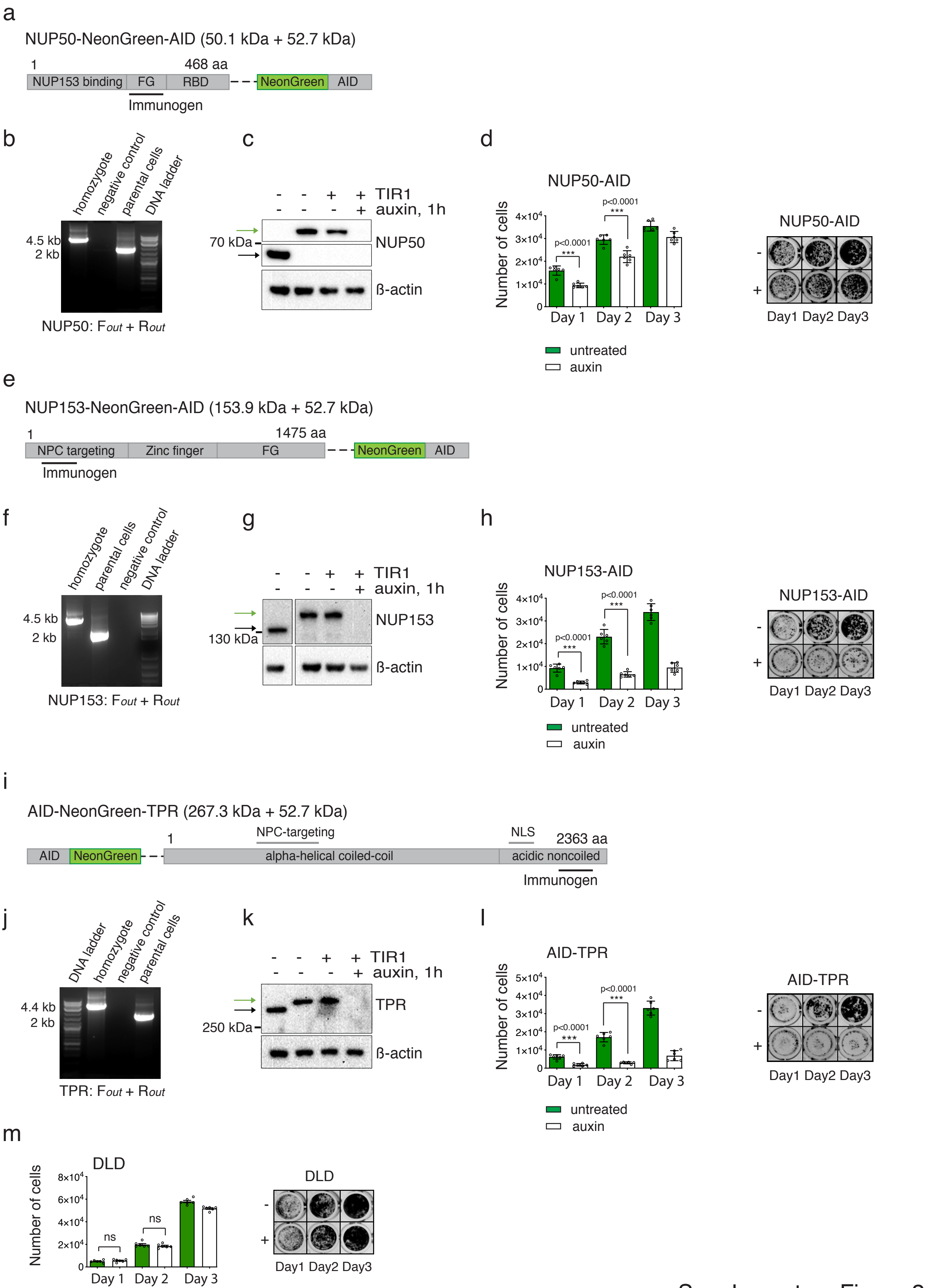

Supplementary Figure 2

Supplementary Figure 2. CRISPR/Cas9 targeting of BSK-NUPs. a, e, i, Regions of NUP50, NUP153, and TPR used for antibodies production (Bethyl Laboratories). **b, f, j,** Genomic PCR of homozygous clones demonstrating the integration of the NG-AID-P2A-Hygromycin sequence into genomic loci of *NUP50*, *NUP153*, and *TPR* genes. **c, g, k,** Degradation of NUP50 (**c**), NUP153 (**g**), and TPR (**k**) after 1 h of auxin treatment. Black and green arrows indicate the molecular weight of unmodified and AID-NeonGreen (AID-NG) tagged nucleoporins. **d, h, l,** Growth rate of DLD-1 cells upon loss of NUP50, NUP153, and TPR. Without auxin, AID-tagged cell lines were viable and did not show any obvious defects. Auxin addition led to rapid degradion of AID-tagged proteins and growth arrest of AID-NUP153 and AID-TPR cells in the continuous presence of auxin. Each bar plot represents a mean value of the cell number measurements from six independent wells from one representative experiment. Error bars are SD. p-value *** < 0.0001 (unpaired two-tailed Student's *t*-test). **m**, Growth rate of DLD-1 cells in the absence or presence of auxin. Data are expressed as mean value ± SD, n=6, ns – non-significant (p-value > 0.05, unpaired two-tailed Student's *t*-test). Exact p-values are indicated on the graphs and in Source Data file. Each bar plot represents a mean value of the cell number measurements from six independent wells from one representative experiment. AID – Auxin Inducible Degron, NG – NeonGreen, FG – phenylalanine(Phe)-glycine(Gly), RBD - Ran-binding domain, NLS – Nuclear Localization Signal.

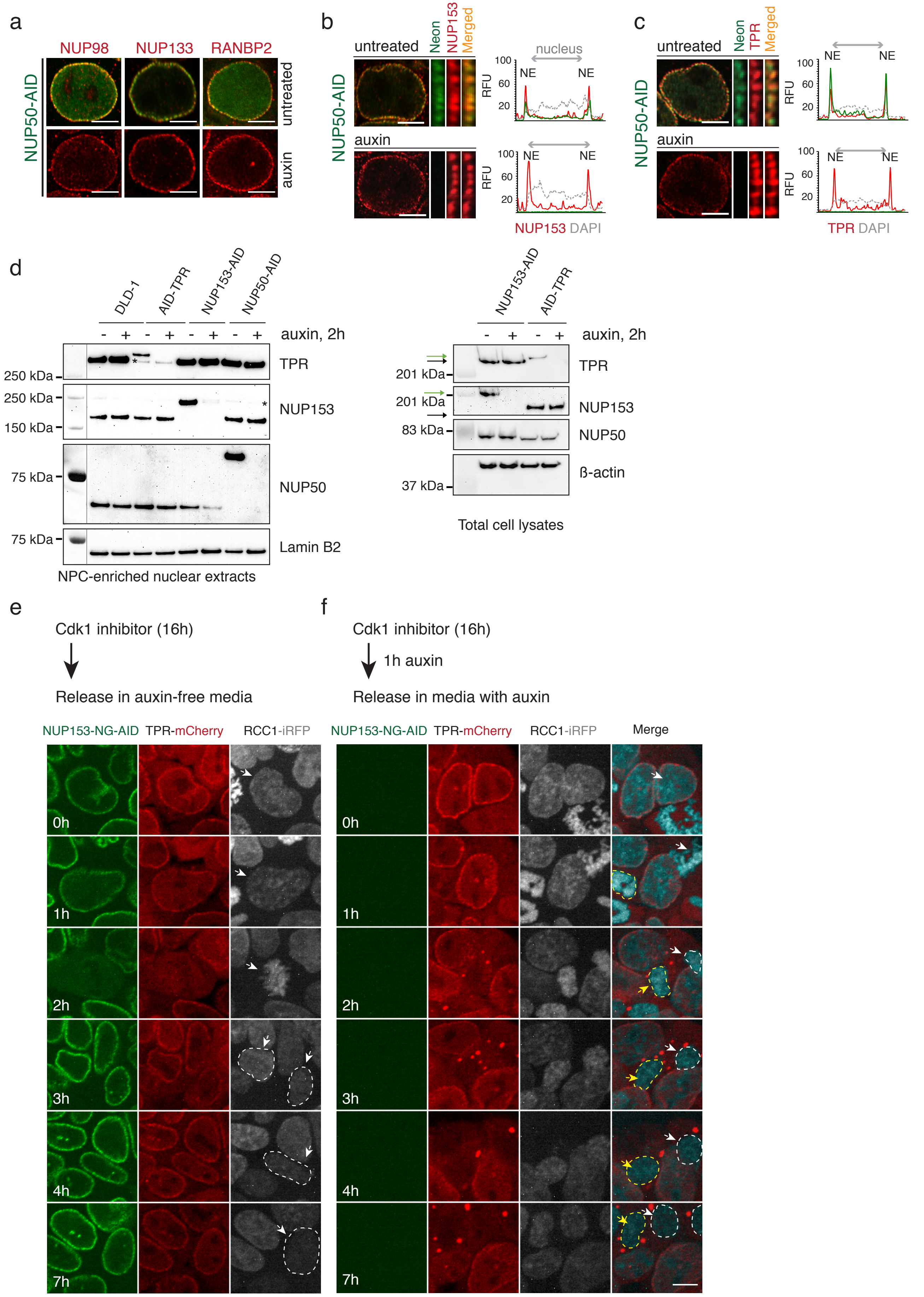

a
NUP98
NUP133
RANBP2
NUP50-AID
untreated
auxin
b
untreated
Neon
NUP153
Merged
nucleus
NE
NE
RFU
100
60
20
auxin
NUP153 DAPI
c
TPR
TPR DAPI
d
DLD-1
AID-TPR
NUP153-AID
NUP50-AID
auxin, 2h
TPR
NUP153
NUP50
Lamin B2
250 kDa
150 kDa
75 kDa
NPC-enriched nuclear extracts
201 kDa
83 kDa
37 kDa
ß-actin
Total cell lysates
e
Cdk1 inhibitor (16h)
Release in auxin-free media
NUP153-NG-AID
TPR-mCherry
RCC1-iRFP
0h
1h
2h
3h
4h
7h
f
1h auxin
Release in media with auxin
Merge

Supplementary Figure 3. **Stability of the assembled nuclear pores after rapid loss of BSK-NUPs**. **a**, Localization of NUP98, NUP133, and RANBP2 nucleoporins in the absence or presence of NUP50 (4 h of auxin treatment). **b**-**c**, Localization of NUP153 (**b**) and TPR (**c**) in the absence or presence of NUP50 (4 h of auxin treatment). Scale bar: 5 μm. **d**, Western blotting of NPC-enriched nuclear extracts and total cell lysates of NUP153-, TPR- and NUP50- depleted cells. Note that loss of TPR (fourth lane) or NUP50 (eighth lane) from the nuclear envelope did not mislocalize other basket nucleoporins. NUP153 depletion results in mislocalization of NUP50 but not TPR from the NPC (Fig.1d, Supplementary Fig.4h). Loss of NUP153 or TPR did not change the total protein amount of other basket nucleoporins in the cells. **e**, **f**, Localization of mCherry-tagged TPR in untreated and NUP153-depleted cells after mitotic exit. Cells were synchronized with Cdk1 inhibitor for 16 h, followed by release in auxin-free media (e) or media supplied with 1 mM auxin (f) and TPR localization in dividing cells was followed for 7 h. To monitor mitotic cells, infrared fluorescent protein (iRFP670) was integrated into the endogenous locus of *RCC1* gene (RCC1-iRFP, marked in grey). Note that post-mitotic NUP153-depleted cells were characterized by the smaller size of their nuclei for prolonged time and TPR-mCherry was neither imported to the nucleus nor integrated into the nuclear envelope, but rather resides as cytoplasmic aggregates. Scale bar: 5 μm. RFU - Relative fluorescence intensity, NE – nuclear envelope.

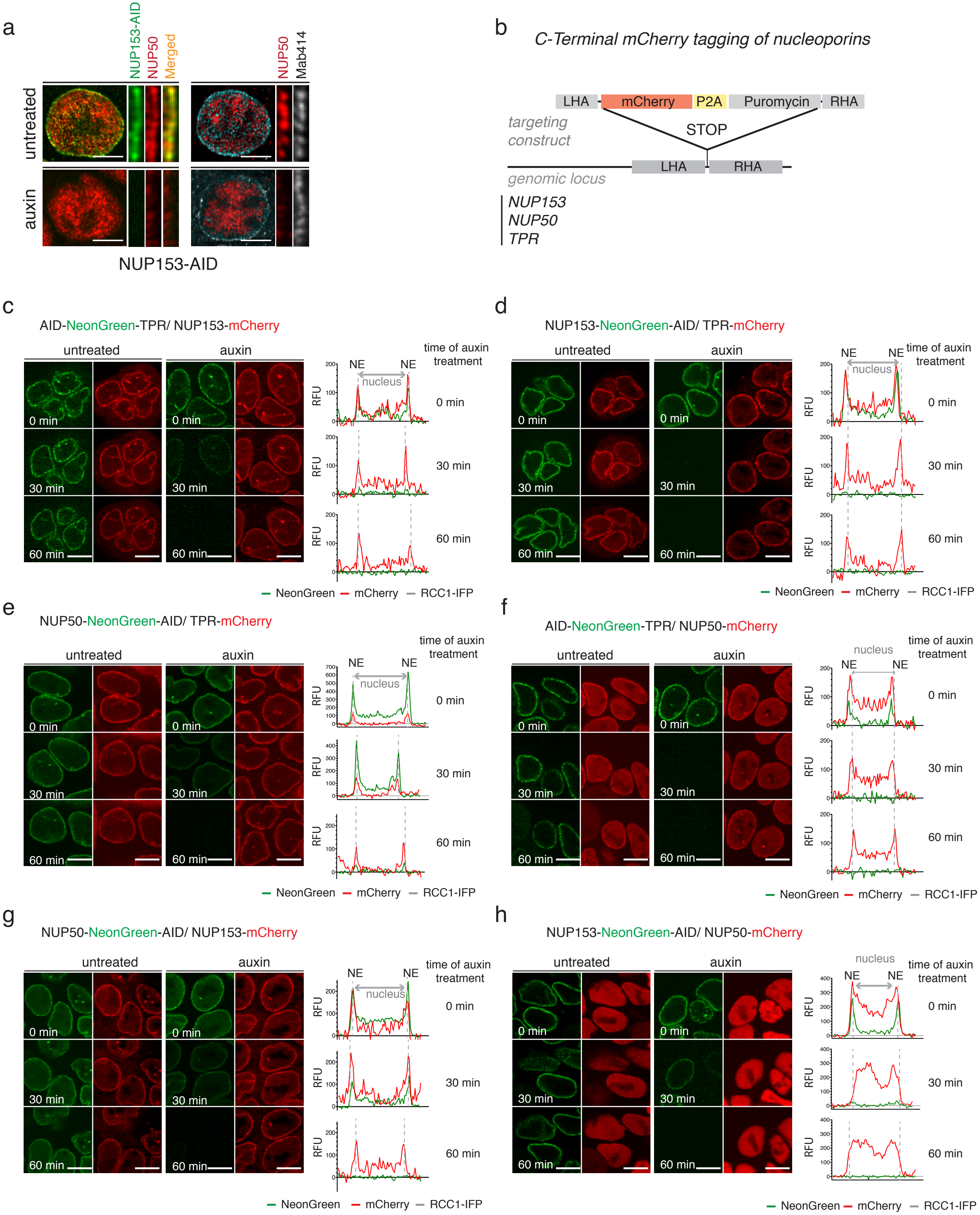
a
NUP153-AID
NUP50
Merged
NUP50
Mab414
untreated
auxin
NUP153-AID
b
C-Terminal mCherry tagging of nucleoporins
LHA
mCherry
P2A
Puromycin
RHA
targeting construct
STOP
LHA
RHA
genomic locus
NUP153
NUP50
TPR
c
AID-NeonGreen-TPR/ NUP153-mCherry
d
NUP153-NeonGreen-AID/ TPR-mCherry
e
NUP50-NeonGreen-AID/ TPR-mCherry
f
AID-NeonGreen-TPR/ NUP50-mCherry
g
NUP50-NeonGreen-AID/ NUP153-mCherry
h
NUP153-NeonGreen-AID/ NUP50-mCherry
untreated
auxin
0 min
30 min
60 min
NE
nucleus
RFU
time of auxin treatment
NeonGreen
mCherry
RCC1-IFP

Supplementary Figure 4. Supporting data regarding interdependence of BSK-NUPs. **a**, Co-localization of NUP50 with NUP153 (green, left) or Mab414-positive nucleoporins (grey, right) in the absence or presence of NUP153 (4 h of auxin treatment, scale bar: 5 µm). NUP153 loss leads to NUP50 re-localization from the NE to nucleoplasm. **b**, A scheme of mCherry integration into genomic loci of *NUP153*, *NUP50*, and *TPR* genes. **c**-**h**, mCherry-tagged NUP153, NUP50 or TPR were integrated into corresponding AID-BSK-NUPs cell lines and were used to follow NUP153-TPR-NUP50 interdependency. Representative images of mCherry signal at 30 and 60 min after auxin addition are shown in red. Scale bar: 10 µm. RFU - Relative fluorescence intensity, NE – nuclear envelope, LHA – left homology arm; RHA – right homology arm.

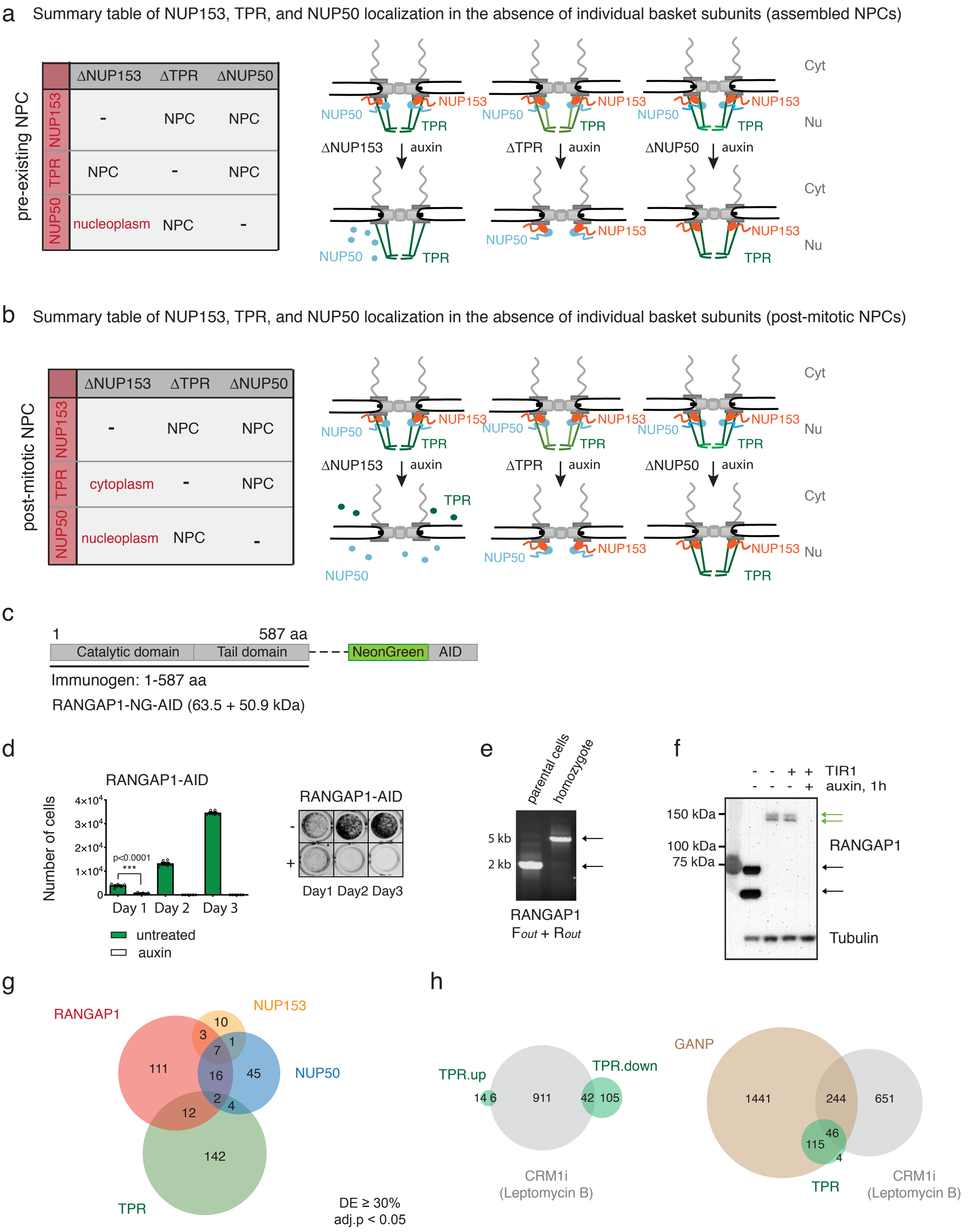
a
Summary table of NUP153, TPR, and NUP50 localization in the absence of individual basket subunits (assembled NPCs)
pre-existing NPC
ΔNUP153 ΔTPR ΔNUP50
NUP153 - NPC NPC
TPR NPC - NPC
NUP50 nucleoplasm NPC -
NUP153
NUP50
TPR
ΔNUP153 auxin
ΔTPR auxin
ΔNUP50 auxin
Cyt
Nu
b
Summary table of NUP153, TPR, and NUP50 localization in the absence of individual basket subunits (post-mitotic NPCs)
post-mitotic NPC
ΔNUP153 ΔTPR ΔNUP50
NUP153 - NPC NPC
TPR cytoplasm - NPC
NUP50 nucleoplasm NPC -
c
1
587 aa
Catalytic domain
Tail domain
NeonGreen
AID
Immunogen: 1-587 aa
RANGAP1-NG-AID (63.5 + 50.9 kDa)
d
RANGAP1-AID
Number of cells
4x10^4
3x10^4
2x10^4
1x10^4
0
p<0.0001
***
Day 1 Day 2 Day 3
untreated
auxin
RANGAP1-AID
-
+
Day1 Day2 Day3
e
parental cells
homozygote
5 kb
2 kb
RANGAP1
Fout + Rout
f
TIR1
auxin, 1h
150 kDa
100 kDa
75 kDa
RANGAP1
Tubulin
g
RANGAP1
NUP153
NUP50
TPR
10
3
1
7
111
16
45
2
4
12
142
DE ≥ 30%
adj.p < 0.05
h
TPR.up
TPR.down
14 6
911
42 105
CRM1i
(Leptomycin B)
GANP
1441
244
651
46
115
4
TPR
CRM1i
(Leptomycin B)

Supplementary Figure 5. **Summary Tables of interdependence of basket nucleoporins.** Analysis of BSK-NUPs interdependence in interphase of pre-existing NPC (a) and post-mitotic NPC (b) upon loss of NUP153, TPR, or NUP50. Assembled NPC is stable, and loss of individual basket nucleoporin does not lead to loss of other nucleoporins from the NPC, except the case of NUP50, which requires NUP153 for its localization (Supplementary Data 1). NUP153 does not control localization of TPR in the pre-existing NPC, but is essential for TPR recruitment to NPC after mitotic NPC re-assembly. **c**, Protein regions of RANGAP1 detected by respective antibody. **d**, Growth rates of AID-tagged RANGAP1 cells upon auxin treatment for 1-3 days. Each bar plot represents a mean value of the cell number measurements from six independent wells from one representative experiment. Error bars are SD. p-value *** < 0.0001 (unpaired two-tailed Student's *t*-test). **e**, Genomic PCR demonstrating the integration of the AID degron sequence into the locus of *RANGAP1* gene. **f**, Western blot analysis of RANGAP1-AID cells. Black and green arrows indicate the molecular weight of endogenous and tagged proteins. Note rapid degradation of RANGAP1-AID-tagged protein happen within 1 h. **g**, Venn diagram representing the number of RNAs that showed significant change (both up- and down-regulation) upon NUP50 (blue), NUP153 (yellow), TPR (green), and RANGAP1 (red) loss. For the visualization purpose the following overlaps are not shown: NUP153-NUP50-TPR (1 gene), NUP153-TPR-RANGAP1 (1 gene), NUP153-NUP50-TPR-RANGAP1 (2 genes). **Functional relationship between TPR/GANP-dependent and CRM1-dependent transcripts. h** (left), A Venn diagram representing the overlap of up- and down-regulated TPR-dependent genes with CRM1-dependent genes. This overlap is not significant: Representation factor: 0.3, p < 1.271e-27, Hypergeometric distribution test. **h** (right), A Venn diagram representing the overlap between RNA transcripts whose expression was affected by acute loss of either GANP (brown), TPR (green) or CRM1 (grey). TPR and GANP were controlled by auxin-AID system (2 or 3 h of auxin treatment, respectively); CRM1 was inhibited by Leptomycin B (LMB, 3 h). DE log2FC>30%, adj. p-value < 0.05, Wald test. Three independent biological replicates were used to perform RNA-Seq of indicated cell lines and LMB-treated DLD-1 cells. DE – Differentially Expressed, NPC – Nuclear Pore Complex.

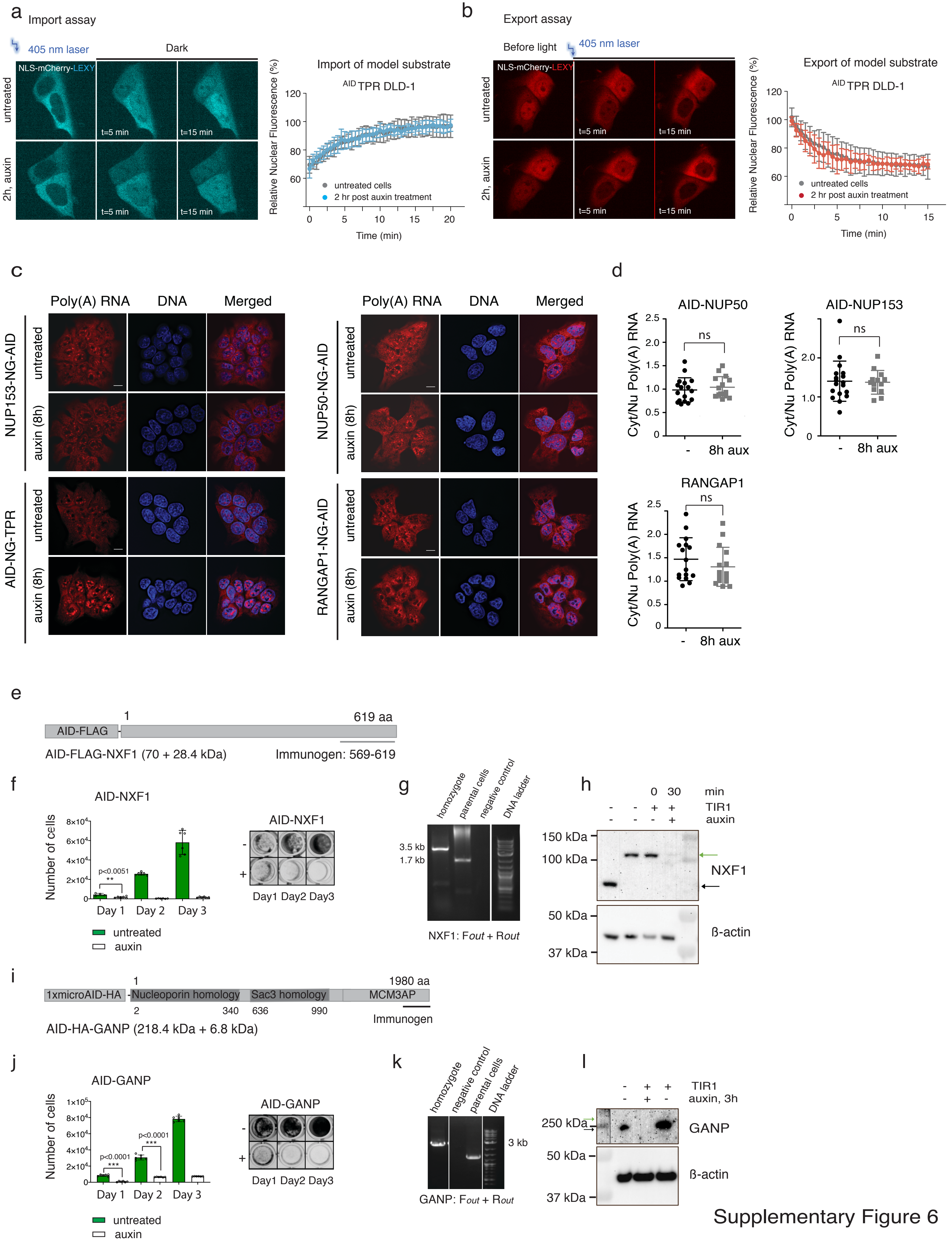

Supplementary Figure 6

Supplementary Figure 6. **Effect of BSK-NUPs loss on the nuclear-cytoplasmic transport. a-b**, Representative images of the import (a) and export (b) of model substrate (NLS-mCherry-LEXY) in AID-TPR cell line. Representative images are shown for untreated cells and 2 h auxin-treated cells before, 5 min and 15 min after blue light (405 nm) exposure for the export assay, or before, 5 min and 15 min after imaging cells in the dark for import assay. For import assay NLS-mCherry-LEXY signal is shown in Turquoise for representation purposes. Both half-life of import (p-value - 0.126) and export (p-value - 0.290) were not significant between untreated cells and after TPR loss. Half-life of untreated vs. auxin treated cells were compared by unpaired two-tailed Student's *t*-test (n=6). Each data point represents as mean value ± SD. **Effect of BSK-NUPs and RANGAP1 depletion on the nuclear-cytoplasmic distribution of poly(A) RNA. c**, Representative images of poly(A) RNA distribution in untreated and auxin (8h) treated NUP153-, TPR-, NUP50- or RANGAP1- NG-AID targeted cells. poly(A) RNA was visualized using oligo(dT)-Quasar 670 probe. Scale bar: 10 µm. **d**, Quantification of cytoplasmic to nuclear (Cyt/Nu) Poly(A) RNA distribution in AID-tagged NUP50, NUP153, RANGAP1, and TPR cells in the presence or absence of auxin 8 h. Biological triplicates from two independent experiments were used for two-tailed Student's *t*-test, ns - non-significant ($p > 0.05$). Data are presented as mean values; error bars are SD. **NXF1, and GANP tagging strategy. e, i**, Protein regions of NXF1 and GANP detected by respective antibodies. **f, j** Growth rates of AID-tagged NXF1 and GANP cells upon auxin treatment for 1-3 days. Each bar plot represents a mean value of the cell number measurements from six independent wells from one representative experiment, except for NXF1 control cells, whose number was n=4. Error bars are SD. p-value ** p=0.0051, *** < 0.0001 (unpaired two-tailed Student's *t*-test). **g**, **k**, Genomic PCR demonstrating the integration of the AID degron sequence into the locus of *NXF1* or *GANP* genes. **h, l** Western blot analysis of AID-NXF1 or AID-GANP cells. Black and green arrows indicate the molecular weight of endogenous and tagged proteins. Note rapid degradation of AID-tagged proteins.

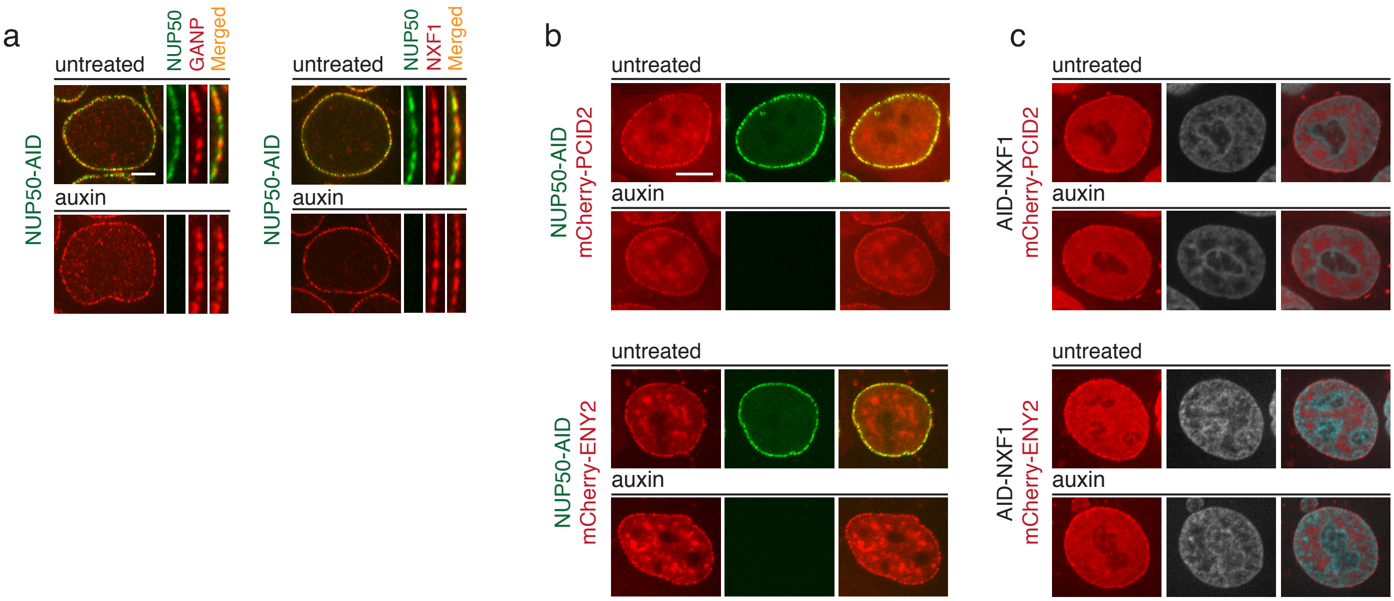

d

Summary table of TREX2 complex subunits localization in the absence of individual basket subunits (post-mitotic NPCs)

pre-existing NPC	ΔTPR	ΔNXF1	ΔGANP	ΔNUP153	ΔNUP50
TPR	-	NPC	NPC	NPC	NPC
NXF1	NPC+Nu	-	NPC+Nu	NPC+Nu	NPC+Nu
GANP	Nu	NPC+Nu	-	NPC+Nu	NPC+Nu
PCID2	Nu	NPC+Nu	Nu	NPC+Nu	NPC+Nu
ENY2	Nu (aggreages)	NPC+Nu	Nu	NPC+Nu	NPC+Nu

e

Top GO-terms, RNAs subgroups (Fig.3 d)*

TPR-GANP-NXF1 genes**	N. matches	p-value
Reg. of NA-templated transcription	38	7.35e-06
Reg. of RNA biosynthetic process	38	7.62e-06
Reg. of cel.biosyn. process	42	1.11e-05

GANP-NXF1 genes	N. matches	p-value
Nucleobase-cont. compound biosyn.	69	1.37e-05
Transcription	63	2.22e-05
RNA biosynthetic process	63	4.37e-05

GANP genes	N. matches	p-value
Chromatin organization	39	1.45e-16
Nucleosome assembly	19	5.24e-14
DNA packing	20	2.96e-12

Protein Domain Enrichment for GANP specific genes

Histone-fold	31	3.24e-36
Histone H2A/H2B/H3	24	1.41e-30
Histone H2B	12	1.61e-16

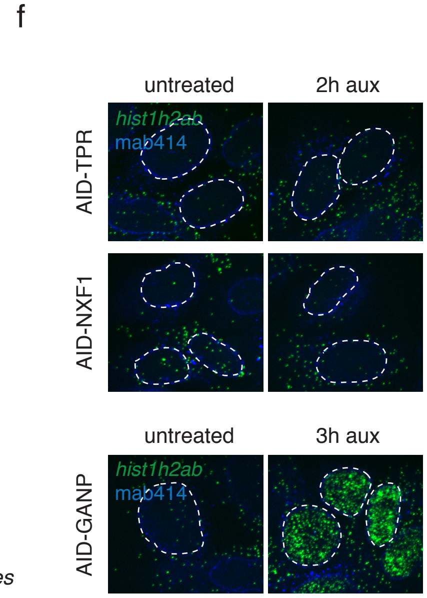

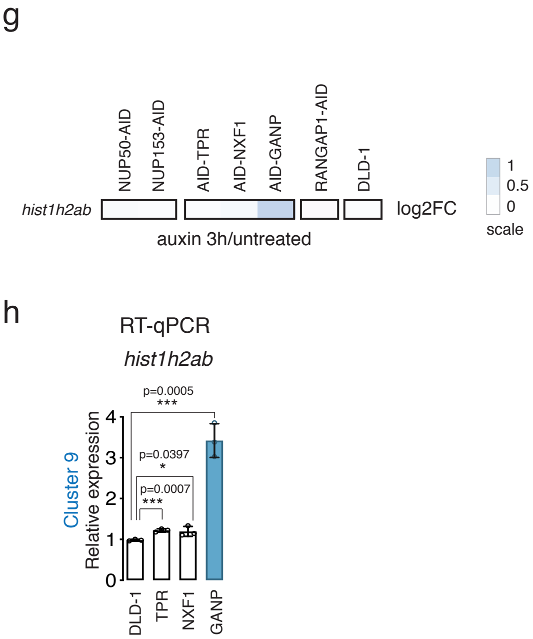

.

Supplementary Figure 7. Data supporting tethering of TREX2 complex subunits to the NPC through TPR. a, Localization of GANP and NXF1 (fixed samples) to the NPC after NUP50 loss. Scale bar is 5 µm. **b**, **c**, Localization of PCID2 and ENY2 (live imaging) subunits to the NPC after NUP50 or NXF1 loss. Scale bar is 7 µm. **d**, A summary table of the interdependence of TPR, NXF1, and TREX2 complex subunits (GANP, PCID2, ENY2) at the NE. TPR but not NUP153 or NUP50 anchors GANP, PCID2, and ENY2 subunits to the nuclear periphery. TPR is required for GANP-NPC localization, and GANP serves as a platform for PCID2 and ENY2 binding. All major changes are indicated in red. **e**, Top GO-terms for TPR-GANP-NXF1 (120 genes), GANP-NXF1 (206), and GANP subgroups (167 genes) (from Venn diagram in Fig.2d, Supplementary Data 3). * HumanMine database v7 2020 was used for GO terms analysis. Transcripts with FC > 0.2 for TPR (GANP-NXF1 group) and both TPR and NXF1 (GANP group) was removed from the analysis to focus on transcripts that specifically changed only upon GANP, GANP-NXF1, or TPR-GANP-NXF1 loss. ** No Holm-Bonferroni test correction was used for Gene Ontology (GO-terms) enrichment analysis. **f**, RNA-FISH analysis of localization of *hist1h2ab* transcript after TPR, NXF1, or GANP loss. *Hist1h2ab* transcript is highly upregulated upon GANP loss but not after TPR depletion. Note 2 h of TPR depletion affects GANP localization on the NE and can affect expression of GANP-specific transcripts at later time points. *Hist1h2ab* transcript shown in green, nuclear envelope signal marked in blue and dashed white line. Data are representative of two independent experiments. **g**, A heat map of log2FC of *hist1h2ab* transcript from RNA-Seq data (Supplementary Data 2), *hist1h2ab* mRNA is significantly upregulated after 3 h of GANP loss (logFC 0.93, p.adj. 4.34e-11). **h**, RT-qPCR analysis of *hist1h2ab* mRNA after 2 h of TPR, NXF1, or 3 h of GANP loss. Graphs show mean values of three technical replicates of one experiment. Error bars are SD. Asterisks indicate p-value * < 0.1, *** < 0.001 (unpaired two-tailed Student's *t*-test). Exact p-value indicated on the graphs and in Source Data file. Reg. – Regulation, NA – Nucleic Acid, Cont. – Containing, Cel.biosyn. – Cellular Biosynthetic, NE – Nuclear Envelope, FC – Fold Change.

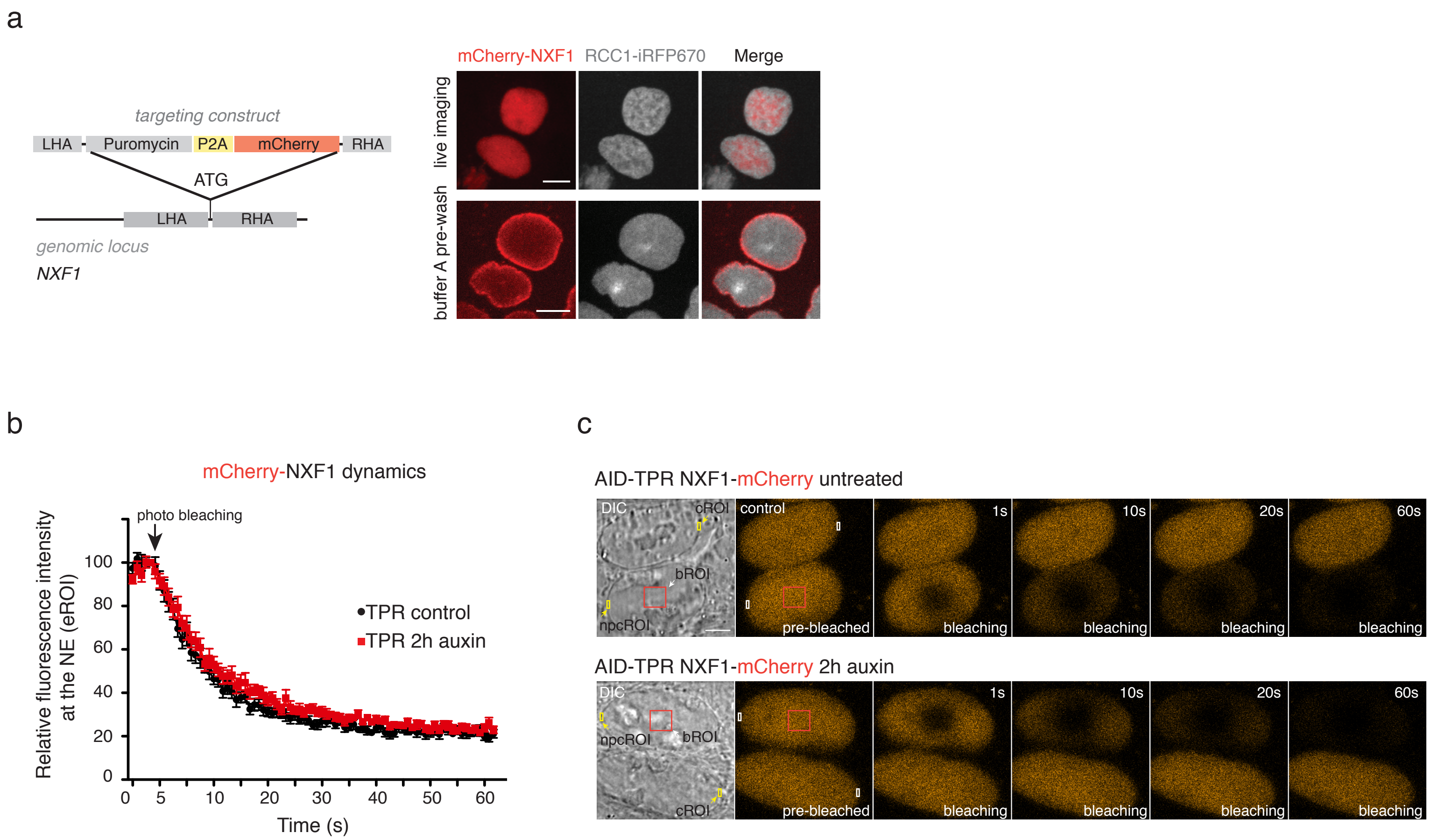

Supplementary Figure 8. Fluorescence loss in photobleaching (FLIP) of mCherry-NXF1 at the NE. a, A schematic of mCherry insertion into NXF1 genomic locus and localization of endogenous NXF1 (mCherry-tagged) in AIDTPR DLD-1 cell line. Most of the NXF1 is localized in the nucleoplasm. NXF1 signal at the NE is not visible in live cells due to dominating signal in the nucleoplasm (upper panel). NPC-bound NXF1 can be visualized after permeabilization of live cells with buffer A (see Materials and Methods, mass-spectrometry section) (lower panel); same protocol was used to obtain images in Figure 3d-g. Scale bar: 10 µm. **b-c**, FLIP experiment. Dynamics of exchange between NPC-bound and nucleoplasmic NXF1 before and after TPR loss. Center of the nucleus (red rectangle) was bleached with 568 laser line and NXF1 fluorescence intensity was measured at the NE region (as assessed by DIC imaging; ROI, yellow rectangle). Continuous photobleaching led to rapid disappearance of NXF1 from all regions within the cell (nucleoplasm and NE) within 30 s. TPR loss did not affect dynamics of NXF1 within the nucleus (unpaired two-tailed Student's t-test, p-value is 0.353, auxin-treated cells n = 17, untreated cells = 19). Data are presented as mean value; error bars are SD. Scale bar: 5 µm. Data and images are representative of three independent experiments. LHA – left homology arm; RHA – right homology arm, NE – Nuclear Envelope, DIC – Differential Interference Contrast, ROI – Region Of Interest, cROI – ROI at the nuclear envelope in control cell, npc – ROI at the nuclear envelope in bleached cell.

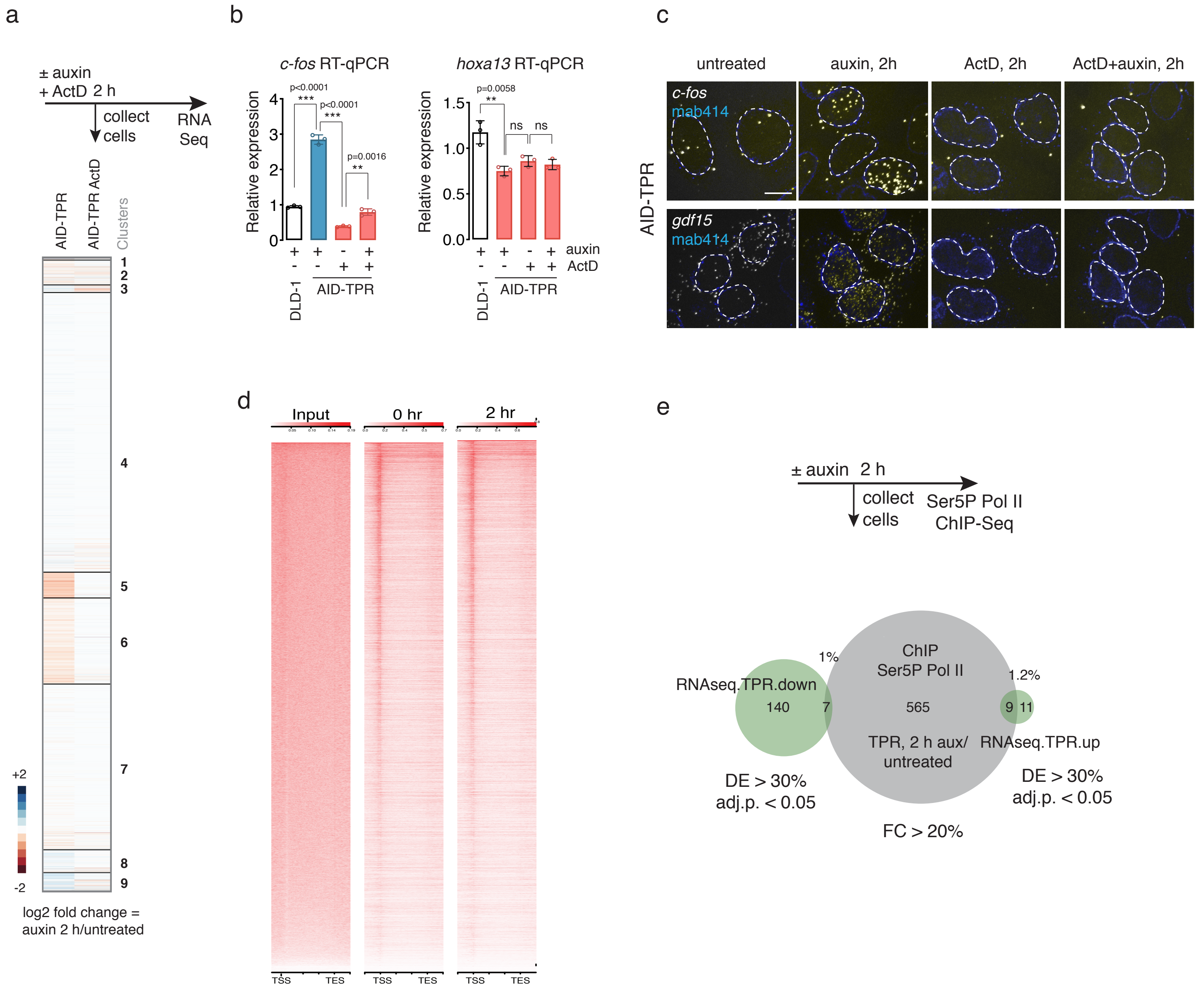
a
± auxin
+ ActD 2 h
collect cells
RNA Seq
AID-TPR
AID-TPR ActD
Clusters
1
2
3
4
5
6
7
8
9
+2
-2
log2 fold change = auxin 2 h/untreated
b
c-fos RT-qPCR
hoxa13 RT-qPCR
Relative expression
p<0.0001
p<0.0001
p=0.0016
p=0.0058
ns
auxin
ActD
DLD-1
AID-TPR
c
untreated
auxin, 2h
ActD, 2h
ActD+auxin, 2h
AID-TPR
c-fos
mab414
gdf15
d
Input
0 hr
2 hr
TSS
TES
e
± auxin 2 h
collect cells
Ser5P Pol II ChIP-Seq
ChIP Ser5P Pol II
RNAseq.TPR.down
140
7
565
9
11
1%
1.2%
TPR, 2 h aux/ untreated
RNAseq.TPR.up
DE > 30%
adj.p. < 0.05
FC > 20%

Supplementary Figure 9. **TPR-regulated RNAs rely on ongoing transcription. a**, A heat map of differential RNA expression upon TPR loss with and without ongoing transcription: Left lane shows a heat map of differentially expressed RNAs upon loss of TPR (2 h auxin vs. untreated). Right lane shows differentially expressed RNAs upon loss of TPR in the presence of Actinomycin D (ActD) (2 h ActD and auxin vs. ActD). Three independent biological replicates were performed for RNA-Seq. **b**, RT-qPCR of *c-fos* and *hoxa13* expression after ActD treatment. Graphs show mean values of three technical replicates of one representative experiment. Error bars are SD. Asterisks indicate only statistically significant p-value ** < 0.01, *** < 0.001 (unpaired two-tailed Student's *t*-test), ns – non-significant (p > 0.05). Exact p-value indicated on the graphs and in Source Data file. **c**, RNA localization and abundance of two top upregulated transcripts (*c-fos* and *gdf15*) 2 h after TPR loss. ActD treatment abolished transcription of *C-FOS* and *GDF15* genes in ActD-treated cells with or without TPR. **d**, ChIP of RNA Pol II Ser5P of AID-TPR cells in the absence (0 h) and presence (2 h) of auxin. A heat map of the aligned regions (± 2 kb) from transcription start site (TSS) and transcription end site (TES) for all genes. Input samples did not show RNA Pol II Ser5P enrichment at the TSS or TES sites. **e**, A Venn diagram of Ser5P Pol II enriched genes and differentially expressed RNAs 2 h after TPR loss. Pol II occupancy around the transcription start sites (TSSs) and gene body was changed only for 16 differentially expressed TPR-specific genes (9 up- and 7 downregulated genes). All 9 upregulated and 4 downregulated transcripts display correlation to RNA-Seq data. Two independent biological replicates were performed for ChIP-seq analysis.

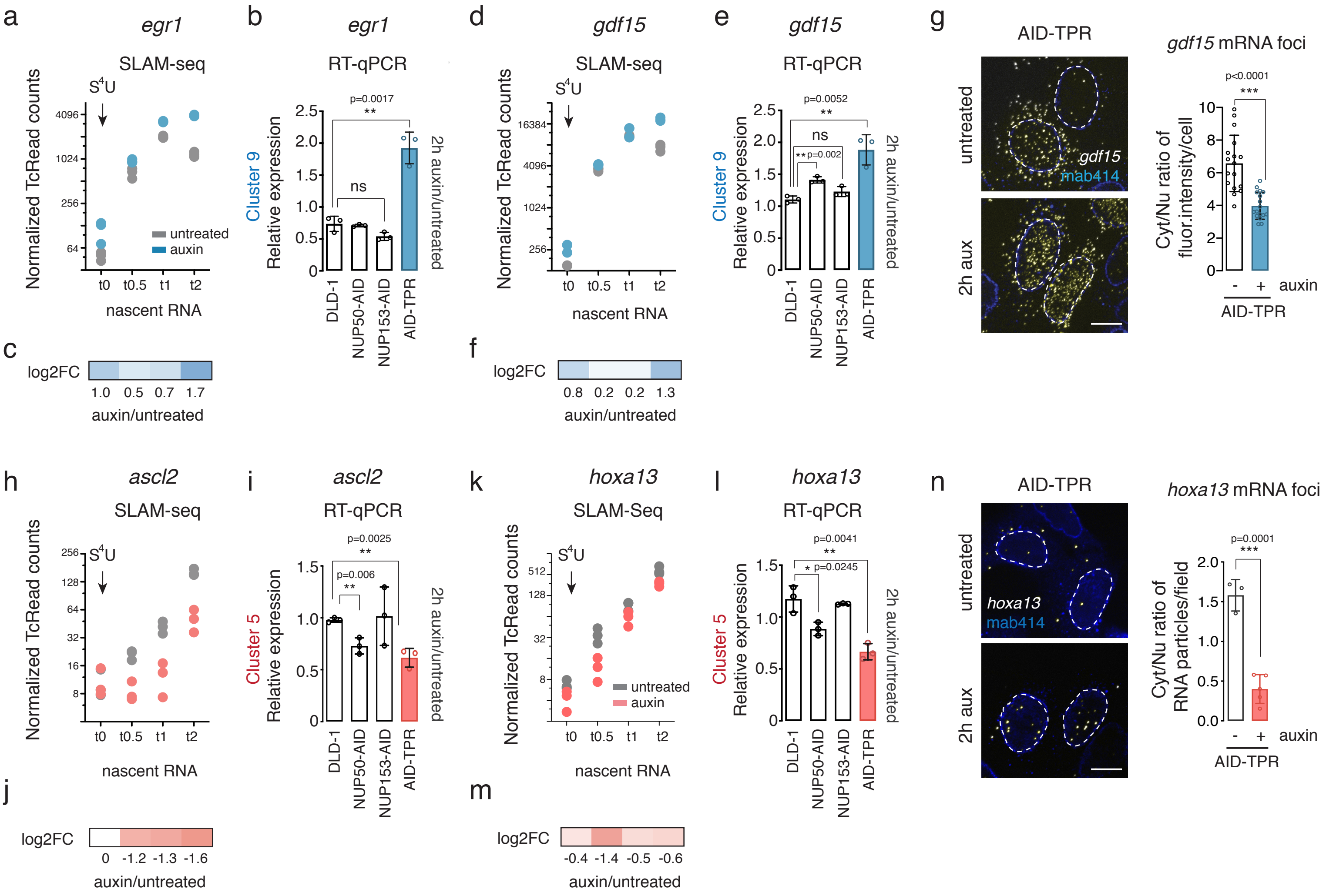

Supplementary Figure 10. Supporting data for transcription and RNA export of TPR-specific RNAs. a, d, h, k, Normalized T>C count plots of additional top targets: *egr1* (a), *gdf15* (d), *ascl2* (h) and *hoxa13* (k) 2 h after TPR loss and 0 h, 0.5 h, 1 h, and 2 h after subsequent S4U pulse. Three independent replicates are shown on the plot. **b, e, i, l** qRT-PCR data supporting RNA-seq changes in RNA abundance of *egr1*, *gdf15*, *ascl2*, and *hoxa13* transcripts 2 h after TPR, NUP50, or NUP153 loss. Graphs show mean values of three technical replicates of one representative experiment. Error bars are SD. Asterisks indicate p-value * < 0.1, ** < 0.01, *** < 0.001 (unpaired two-tailed Student's t-test), ns – non-significant ($p > 0.05$). Exact p-value indicated on the graphs and in Source Data file. For all plots, auxin treated samples were compared to untreated cells. **c, f, j, m,** Log2FC of T>C counts of auxin treated vs. untreated samples were used to show changes in the abundance of the transcripts at each time point from three independent biological replicates (Supplementary Data 5). **g, n,** Localization of *gdf15* and *hoxa13* RNAs after TPR loss, assayed by fluorescence *in situ* hybridization (FISH). Note that *hoxa13* mRNA levels decreased but remaining mRNA foci were localized inside the nucleus; for *gdf15* we registered both an increase of the number of cells with the higher level of *gdf15* and accumulation of *gdf15* transcripts within the nucleus. To quantify *hoxa13* transcripts, we analyzed discrete mRNA foci in the nucleus and the cytoplasm from three (untreated) and five (auxin treated) independent fields of AID-TPR cell line (***p-value 0.0001). To quantify *gdf15* mRNA distribution, fluorescence intensity in the cytoplasm was compared to the nucleus (untreated cells, n=17; auxin treated cells, n=17, ***p-value < 0.0001). Data are presented as mean values; error bars are SD (unpaired two-tailed Student's *t*-test). Data are representative of two independent experiments. Nuclear envelope signal marked in blue (Mab414) and dashed white line. FC – Fold Change.

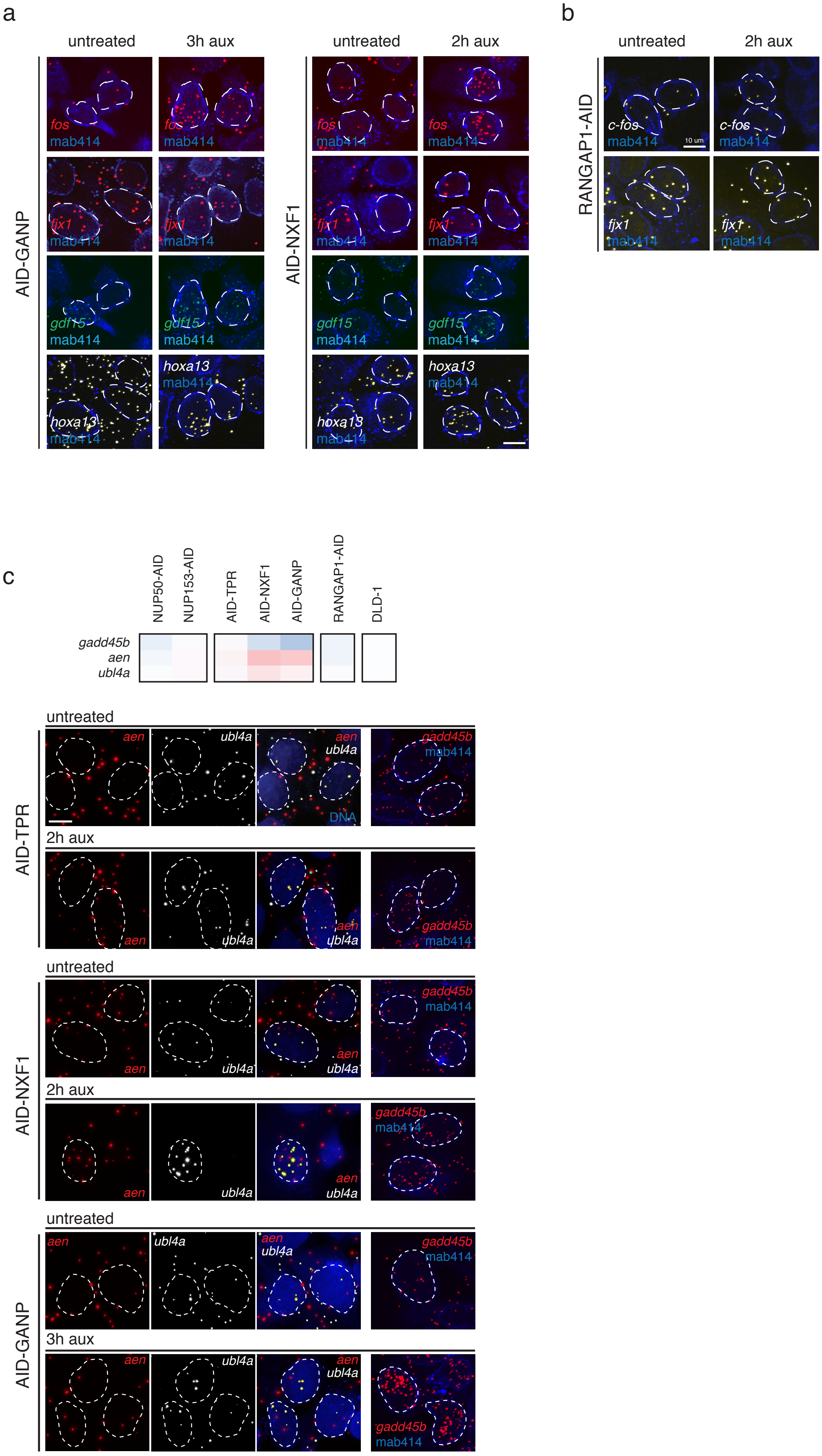
a
untreated
3h aux
untreated
2h aux
AID-GANP
AID-NXF1
fos
mab414
fjx1
mab414
gdf15
mab414
hoxa13
mab414
b
untreated
2h aux
RANGAP1-AID
c-fos
mab414
10 um
fjx1
mab414
c
NUP50-AID
NUP153-AID
AID-TPR
AID-NXF1
AID-GANP
RANGAP1-AID
DLD-1
gadd45b
aen
ubl4a
untreated
2h aux
3h aux
AID-TPR
AID-NXF1
AID-GANP
aen
ubl4a
DNA
gadd45b
mab414

Supplementary Figure 11. TPR regulates RNA localization and abundance of GANP-dependent transcripts. **a**, Localization of *c-fos*, *fjx1, gdf15,* and *hoxa13* RNAs after 3 h of GANP and 2 h of NXF1 loss. **b**, *c-fos* and *fjx1* RNA abundance is not changed upon RANGAP1 loss. Scale bar 10 µm. **c**, RNA localization of GANP-NXF1-specific transcripts, *aen*, *ubl4a*, and *gadd45b*. Corresponding transcripts were down- or upregulated upon NXF1 or GANP but not TPR loss. All RNA transcripts were enriched within the nucleus upon NXF1 or GANP loss. Note 2 h of TPR depletion affects GANP localization on the NE and can affect expression of GANP-specific transcripts at later time points. Scale bar: 10 µm.

Supplementary Table 1. Oligonucleotides

Gene	Oligonucleotides	PCR product length	Annealing Temp (℃)
Amplification of homology arms			
NUP50	FNUP50 LH aaaacatatgTGAGTCCCAGGATAATAGTGTGTAAG	938 bp	60
NUP50	RNUP50 LH aaaaGATATCggcatcctttttctccagtaaaattttgtgGAGTTCATCAGCGTCTTCGGAGGTTTTTACCCGAATCAACATGGT		
NUP50	FNUP50 RH aaaactcgagCAGAATTATTGCCAAGTTGCTGCTG	878 bp	62
NUP50	RNUP50 RH aaaagcggccgcTTGGTCACAGTTTTCATGTGCGT		
NUP153	FNUP153 LH aaaacatatgTGCAGTGGCTTACGCCTGTA	933 bp	62
NUP153	RNUP153 LH aaaaGATATCctttctccgTCTAACAGCAGTCTTTATCTTGCGAC		
NUP153	FNUP153RH aaaactcgagTCACATTGGTGTTGTACTCAATT	819 bp	57
NUP153	RNUP153RH aaaagcggccgcGATTTCCAAATGGATTTAGGCAAC		
TPR	FTPRNLH ggaattccatatgAGATTCCCTCCGGTCTCAATTCAC	855 bp	62
TPR	RTPRNLH cgggatccCGCCATGTCGGTGGGGCCAGG		
TPR	FTPRNRH ccgctcgagATGGCggCGGTGTTGCAGCAgGTaCTtGAaCGtACtGAaCTGAACAAGCTGCCCAAGTCTGTC	874 bp	62
TPR	RTPRNRH atagtttagcggccgcTCAACTTAGCAAGAACAGTATGAACC		
TPR	FTPRCLH ggaattccatatgCTTAGATACTAGTAGTAGTCAACCAAAG	690 bp	62
TPR	RTPRCLH ggaagatctGTTGATGTTGCCCCGGTTGATGCCGCCCCGGCCGCCCATGGCGTGGGACACTCCTGTAAAAGAAAAAAGAATCAAATTGAATATATC		
TPR	FTPRCRH ccgctcgagTAAATGGTCTGTAAACAATAACAACTGTGAATAAG	704 bp	62
TPR	RTPRCRH ttttcctttgcggccgcCCAGTTACTAGAGTAAACTTGCTACTG		

RANGAP1	FRANGAP1CLH GCCCATATGggcgcgccTCAGGCGCAGTGGCTCCC	1079 bp	50 (Herculase II)
RANGAP1	RRANGAP1CLH GGATCCGGAcggccggccaACtTTaTACAGaGTCTGCAGCAGAgaaTGcCGGGCaAAaGAaCAaGAcTCCAGGGCGgaaTTaGGtCTGCGGGGAAAGAGGGGT		
RANGAP1	FRANGAP1CRH CGCCTCGAGgtttaaacGCCCTGGAATCCTGCTCCT	1083 bp	54
RANGAP1	RRANGAP1CRH gtcgcggccgcctgcaggGCTGCCACACGACTTTATTTGTGAAG		
RCC1	FRCC1LH ggaattccatatgGGAGGCAATGGGACTGGAACCC	779 bp	64
RCC1	RRCC1LH gaagatctAGACTGCTCTTTGTCCTTGACCAAGAGTACAGTATGCTGACCTCCAGAGCTAACGCTCAGAACAACTCTATTCTCCAGCTGTTTGCCCATCA		
RCC1	FRCC1RH ccgctcgagTGATGAAGCCTCTGAGGGCCTGG	901 bp	63
RCC1	FRCC1RH atagtttagcggccgcCTATATCCTATTTTCTCAGCCACTGTACAAG		
GANP	FGANPLH taccgccatgcattagttattaatAGTGCTTAGCGCTCCAGC	558 bp	60
GANP	RGANPLH tcatgtcgacGTTCATCTTCTGCTCCAATTATTAGAAGG		
GANP	FGANPRH cggtactagtatgaaccctacaaacccattttcagggcagcagccatcagctttcagtGCGTCTTCTAGTAATGTAGGAACAC	472 bp	58
GANP	RGANPRH tgattatgatctagagtcgcggccgcCAGAAGCAATTTGGCTCTGG		
NXF1	FNXF1LH gcagtacatcaagtgtatcatatgCGATGGGTTTCTAGTAGGGGACGC	784 bp	65
NXF1	RNXF1LH cgccatgccacagcgGGCTCAGGCGCTGGCCGC		
NXF1	FNXF1RH cgatgacaaggtcgacatggcggatgaaggaaaaagttattccGGTAAGTGCTGGTCTTCGGGTCG	777 bp	64
NXF1	RNXF1RH tgattatgatctagagtcgcggccgcCTGTGTAGGACTGGCGTCTTAAAAAAGG		
PCID2	FPCID2LH gcagtacatcaagtgtatcatatgcctgcaggctcgggagcg	490 bp	66
PCID2	RPCID2LH tcatggatcccgccatgggagcgccgcc		
PCID2	FPCID2RH atggcacatatcacaatcaatcagtatctgcaacaggtgggtcctagccgggga	561 bp	62
PCID2	RPCID2RH tgattatgatctagagtcgcggccgccatctgtcagacgcatgtaccg		

ENY2	FPCID2LH gcagtacatcaagtgtatcaTTTAGTAGAGACGGGGTTTC	584 bp	54
ENY2	RPCID2LH ttgtactcggtcatggatccgctaacctggACATCAACATGTACTTTCAGa		
ENY2	FPCID2RH acgagctgtacaagctcgagatggtggttagcaaaatgaataaagatgcgcag atgagagcagcgattaaccaaaagcttatagagactggAGAAAGAGAACG GTAAGTAATAG	508 bp	54
ENY2	RPCID2RH tgattatgatctagagtcgcATTAAATGCTATGATGGAACAAG		
Genotyping			
NUP50	FNUP50out CAGAGCGCATTCAGGCCAA	1976 bp	61
NUP50	RNUP50out ATTCACACGGCTCGTGTGTC		
NUP153	FNUP153out AACCTATCATGTTGCGGCCG	1834 bp	61
NUP153	RNUP153out ATTATCCAAAGTCACCACAGTCCAT		
TPR-N	FTPRNout TGGCAGACAATTACCTCGGAC	1900 bp	60
TPR-N	RTPRNout CAAGGTCAATTCTGCTTGACAGT		
TPR-C	FTPRout CGGTTTGTTAAATGTTCCTCATTGT	1811 bp	59
TPR-C	RTPRout CTGCAATTCAATTCTGCTCTAACC		
RANGAP1	FRANGAP1out GATATTGAGGGCAACAACAGCCAG	2103 bp	63
RANGAP1	RRANGAP1out CCGAGACTATCCCGCTTTCAGTGAG		
RCC1	FRCC1in AGCCCTGTGGAGATGATGGG	178 bp	62
RCC1	RRCC1in CAGCTGTCACTGCTTCCCTGTTC		
GANP	FoutGANP GTGGAAGATTAGGCTCCCCG	1464 bp	63
GANP	RoutGANP GGAAAGGTTCGCCCAAAACC		
NXF1	FNXF1out AGTTGCCACTGCTGGTGAG	1729 bp	60
NXF1	RNXF1out CTAGTGGCAGTGCCAGGC		

gRNA cloning

NUP50	gRNANUP50-1 F caccgTGCCTGAACACGCAAAGT
NUP50	gRNANUP50-1 R aaacACTTTGCGTGTTCAGGCAc
NUP50	gRNANUP50-2 F caccgTTCGGGTAAAAACCAGCG
NUP50	gRNANUP50-2 R aaacCGCTGGTTTTTACCCGAAc
NUP153	gRNANUP153-1 F caccgAAGACTGCTGTTAGACGC
NUP153	gRNANUP153-1 R aaacGCGTCTAACAGCAGTCTTc
NUP153	gRNANUP153-2 F caccGTTAGACGCAGGAAATAA
NUP153	gRNANUP153-2 R aaacTTATTTCCTGCGTCTAAC
TPR	gRNATPRN-1 F caccgAGCAAGTCCTGGAGCGCA
TPR	gRNATPRN-1 R aaacTGCGCTCCAGGACTTGCTc
TPR	gRNATPRN-2 F caccgTTCAGCTCCGTGCGCTCC
TPR	gRNATPRN-2 R aaacGGAGCGCACGGAGCTGAAc
TPR	gRNATPRC-1 F caccgAGGTGTGAGCCATGCAAT
TPR	gRNATPRC-1 R aaacATTGCATGGCTCACACCTc

TPR	gRNATPRC-2 F caccGAGAGGAGGAATAAACAG
TPR	gRNATPRC-2 R aaacCTGTTTATTCCTCCTCTC
RANGAP1	gRNA#1:GGATTCCAGGGCGCTGTTGGG
RANGAP1	gRNA#7:TGACCCCTCTTTCCCCGCAGG
RCC1	gRNARCC1-1 F caccGACACAGATAAGACCACA
RCC1	gRNARCC1-1 R aaacTGTGGTCTTATCTGTGTC
RCC1	gRNARCC1-2 F caccgCTTATCTGTGTCCAGCGG
RCC1	gRNARCC1-2 F aaacCCGCTGGACACAGATAAGc
GANP	gRNA-6_GANP_F caccgCCACTGAAAGGATTAGTT
GANP	gRNA-6_GANP_R aaacAACTAATCCTTTCAGTGGc
GANP	gRNA-11_GANP_F caccgAGACGCCGAAAAAGCACT
GANP	gRNA-11_GANP_R aaacAGTGCTTTTTCGGCGTCTc
NXF1	gRNA NXF1 28F caccgATGCCACAGCGAAGATCA
NXF1	gRNA NXF1 28R aaacTGATCTTCGCTGTGGCATc
NXF1	gRNA NXF1 14F caccgCGAGGGGAAGTCGTACAG
NXF1	gRNA NXF1 14R aaacCTGTACGACTTCCCCTCGc
PCID2	gRNA PCID2 2F caccgCATGGGAGCGCCGCCGAA
PCID2	gRNA PCID2 2R aaacTTCGGCGGCGCTCCCATGc
PCID2	gRNA PCID2 7F caccGCTGCAGGTACTGGTTAA
PCID2	gRNA PCID2 7R aaacTTAACCAGTACCTGCAGC
ENY2	gRNA ENY2 1F caccgTTGTTCATCTTGCTAACC
ENY2	gRNA ENY2 1R aaacGGTTAGCAAGATGAACAAc
ENY2	gRNA ENY2 3F caccgCCAAAAGTTGATAGAAAC
ENY2	gRNA ENY2 3R aaacGTTTCTATCAACTTTTGGc

Predesigned qPCR Assays

actb	Hs.PT.39a.22214847
polr2a	Hs.PT.39a.19639531
hoxa13	Hs.PT.58.22565110
fos	Hs.PT.58.15540029
egr1	Hs.PT.58.40805543.g
gdf15	Hs.PT.58.40089589
fjx1	Hs.PT.58.26410723.g
ascl2	Hs.PT.56a.26973465.g
hist1h2ab	Hs.PT.58.26129234.g

Supplementary methods

Plasmid DNA preparation for transfection. For transfection $5*10^4$ cells/well were plated in 24-well plates a day before transfection. Plasmids for transfection were prepared using the NucleoSpin buffer set (Clontech) and VitaScientific columns. Plasmids were not linearized before transfection. Cells for gene targeting were transfected with 500 ng of donor and gRNA plasmids in ratio 1:1 using ViaFect (Promega) transfection reagent according to the manufacturer's instruction. Seventy-two hours after transfection, cells were seeded on 10-cm dishes with the selective antibiotics (hygromycin 200 µg/ml, blasticidin 10 µg/ml or puromycin 3 µg/ml) until clones were formed on a plate. Analysis of localization and expression of targeted proteins in clones were performed 14-20 days post transfection on a 24-well lumox (Sarstedt) plates. Clones with proper protein localization were propagated for genomic PCR and Western blot analysis in regular complete media without selective antibiotics. After gene targeting only bright, healthy growing clones were propagated for downstream analysis.

Plasmid construction. For gRNA selection we used CRISPR Design Tools from http://crispr.mit.edu:8079 and https://figshare.com/articles/CRISPR_Design_Tool/1117899. pEGFP-N1 vector (Clontech) was used to create a universal donor vector (pCassette). Briefly, the pEGFP-N1 vector was restricted with Nde1 and Not1 enzymes (NEB), and a new multiple cloning sites were inserted into the vector instead of sequences corresponding to the CMV promoter and EGFP protein. The sequences of homology arms were amplified from genomic DNA extracted from DLD-1 cells, the full-length 229-amino acid AID degron (flAID), hygromycin, and TIR1 sequence were amplified by PCR from pcDNA5-EGFP-AID-BubR1 (Addgene #47330)[1] and pBABE TIR1-9Myc (Addgene #47328)[1] plasmids, respectively. The DNA sequence of

NeonGreen fluorescent protein, FLAG tag, HA tag, a minimal functional AID tag (1xmicroAID) 71-114 amino-acid[2], and three copies of reduced AID tag (3xminiAID) 65-132 amino-acid[3] were codon optimized and synthesized (IDT). TPR, NUP153, NUP50, NXF1 were tagged with flAID, RANGAP1 with 3xminiAID and GANP with 1xmicroAID tags (Supplementary Fig. 2, 5, 6). A separate cell line was generated with 1 microAID-HA tag inserted into TPR locus to perform experiments where green channel (Alexa488) was occupied. cDNAs of mCherry and puromycin resistance were amplified from pmCherry-N1 (632523, Clontech) and pICE vector (Addgene #46960)[4], respectively. All PCR reactions were performed using Hi-Fi Taq (Invitrogen) or Herculase II Fusion (Agilent) DNA polymerases with primers listed in Supplementary Table 5.

Crystal violet staining. In brief, suspension of 4-5*10^3 cells/well was plated in twelve independent w ells of 96-well plate. After cells were attached to the plastic, fresh media with 1 mM auxin was added into the corresponding wells, followed by incubations for 0 h, 24 hrs, 48 hrs, and 72 hrs. Zero-hour time point was used for cell number normalization between different cell lines. Cells were washed with PBS, fixed with 70% ethanol for 15 min, stained with 0.1% crystal violet for 15 min, and washed four times in a stream of distilled water. A hundred microliters of 10% acetic acid were added to each well with cells and incubated for 15 min at RT. Measurement of optical density was performed on EnSpire Plate Reader (Perkin Elmer) at 570 nm (OD_{570}). To calculate the number of survived cells, we plotted the calibration curve with the estimated number of cells and measured their OD_{570}.

Analysis of NPC and NPC-associated proteins by mass spectrometry. Cells were seeded onto 150 mm plates and processed once they reach 90% confluency. Nocodazole (5 μg/ml) and

Cytochalasin B (10 µg/ml) were added for 30 min and cells were washed four times with PBS. Cells were then incubated 2 x 5 min in 30 ml buffer A (20 mM HEPES, pH 7.8; 2 mM DTT, 10% sucrose, 5 mM $MgCl_2$, 5 mM EGTA, 1% TX100, 0.075% SDS) at RT, followed by incubation in 15 ml buffer B (20 mM HEPES, pH 7.8; 2 mM DTT, 10% sucrose, 0.1 mM $MgCl_2$), containing 4 µg/ml RNase A (Promega) for 10 min at 37°C , and washed in buffer A. NPCs and associated proteins were eluted by incubating the plates in 12 ml of buffer C (20 mM HEPES, pH 7.8; 150 mM NaCl, 2 mM DTT, 10% sucrose, 0.3% Empigen BB) for 10 min at 37°C. The supernatant was transferred to 14 ml polypropylene tubes and span for 3 min at 28 000g (HB-6 rotor) at 4°C. The supernatant was transferred to the new tubes, incubated on ice for 30 min, and saturated TCA was added to the final concentration of 8%. Tubes were vigorously mixed and incubated on ice for another 30 min. Precipitated protein complexes were sedimented by centrifugation at 28 000 g (HB-6 rotor) for 20 min at 4°C. The supernatant, containing some Empigen-TCA floating complexes, was aspirated. The protein pellet was resuspended in 1.5 ml cold (-20°C) ethanol, vigorously vortexed to solubilize Empigen-TCA complexes, transferred to eppendorf tubes and spun for 15 min at 11 000 g at 4°C using bucket rotor. The pellet was resuspended in ethanol and spun again for 1 min at 11 000 g. This wash was repeated twice. The NPCCF pellet was then solubilized in 100 µl of buffer D (8 M urea, 5 mM DTT) by pipetting, rigorously vortexed and centrifuged for 1 min at 11 000 g. The supernatant was collected, and the pellet was again solubilized in 100 µl of buffer D, vortexed and spun as above. Both supernatants were combined (200 µl total), and the protein concentration was measured. Samples were stored at -70°C before processing for mass spectrometry or SDS-PAGE.

For mass spectrometry analysis, 100 µg of NPC-enriched fraction was diluted in 50 µl of 8 M urea, supplemented with freshly added DTT (20 mM final) and incubated for 1 h at 37°C.

Iodoacetamide (freshly made stock solution in 25 mM ammonium bicarbonate) was then added (50 mM final) and alkylation proceeded for 1 h at RT. The reaction was quenched by 50 mM DTT and samples were diluted 10 x with 25 mM ammonium bicarbonate. Three microgram of trypsin (v5111, Promega) was added and digestion reaction proceeded overnight at 37°C. Samples were acidified by formic acid, and peptides were desalted with Waters Oasis HLB 1cc columns. Peptides were eluted with 1 ml of buffer E (0.1% formic acid in 50% acetonitrile) and dried using SpeedVac. Samples were either individually analyzed with LCMS for label-free quantitation or analyzed as multiplex sets, after labeling with TMT reagents (TMT10plex label reagent set, Thermo Scientific), according to manufacturer's instructions. To increase the protein coverage, each set of pooled TMT samples was separated into 24 fractions using basic reverse phase liquid chromatography (bRPLC)[5].The mass spectrometric analysis was conducted on an LTQ Orbitrap Lumos (Thermo Fisher Scientific) based nanoLCMS system. Briefly, the label-free samples were analyzed with a 3 h gradient at a 120K resolution for MS1 scans and 30K resolution for MS2 scans at 30% HCD energy. Each TMT fraction was analyzed with a 2 h gradient at 120k resolution for MS1 and 50K for MS2 at 38% HCD energy.

Protein identification and quantitation analysis were carried out on Proteome Discoverer 2.2 platform (Thermo Fisher Scientific). Peptide IDs were assigned by searching the resulting LCMS raw data against UniProt/SwissProt Human database using the Mascot algorithm (V2.6, Matrix Science Inc.). And peptide-spectrum matches (PSM) were further validated with Percolate algorithm. Peptides with high confidence (<1% FDR) were filtered for protein identification. Label-free quantitation was based on areas under peptide precursor traces (MS1) that were aligned with the Minora feature detection function in PD2.2. For TMT sets, report ion intensities of ten channels were normalized at the total peptide amount level. Then relative quantitation of individual

proteins was calculated using the normalized report ion intensities of unique peptides with <1% FDR and >30% isolation purity.

We determined the protein-level fold changes based on the median of peptide-level fold changes from the Proteome Discoverer-produced abundances in handling both TMT and Label-free results. Peptides that could be mapped to multiple proteins by Mascot were removed. We also discarded all keratin-related peptides based on the UniProt annotation. We separated peptides that mapped onto UniProt ID P52948 into NUP96 and NUP98, according to their mapping locations. Peptides that are mapped to amino acids from 1 to 880 were counted for NUP96; the others were used for NUP98. To minimize the batch effect, we used the quartile normalization before calculation of fold changes in the Label-free quantification. Such fold changes were visualized after k-means clustering. In determining the optimal k parameter for clustering, we used "mclust" package to calculate Bayesian Information Criterion of the expectation-maximization model[6]. The quartile normalization, k means clustering, and data visualization were performed under R development environment (R Core Team, 2018).

Protein transport assay. ^{AID}TPR cell lines were seeded on Ibidi μ-Slide four well glass bottom slides in complete DMEM media. Cells were then changed to FluoroBrite DMEM (ThermoFisher) media and transfected with 500 ng of NLS-mCherry-LEXY plasmid (pDN122) (Addgene plasmid # 72655[7]) using ViaFect (Promega) transfection reagent according to the manufacturer's protocol. After transfection, cells were kept in the dark for 24 h prior to imaging. NLS-mCherry-LEXY positive cells were first excited with a 561 nm (20% of power was applied) laser line with 30 ms exposure and imaged every 30 seconds for 10 minutes. Next, the cells were exposed to 405 nm (20% of power was applied) laser line with 1 s exposure every 30 seconds to induce nuclear export of model substrate, which was monitored for 15 minutes. 405 nm laser was

then shut off to induce nuclear import of model substrate, which was monitored for 20 minutes. The cells were then exposed to 405 nm laser again to induce another round of nuclear export of model substrate for 15 minutes. During the course of the experiment, cells were imaged every 30 seconds using 561 nm laser to follow mCherry signal of the model substrate.

Image analysis was performed on Volocity (PerkinElmer) and ImageJ (National Institutes of Health) software with Time Series Analyzer V3 plugin and ROI Manager dialog box. We measured fluorescent intensity from three points in both the cytoplasm and the nucleus of each cell to obtain the average intensity. Adjusted nuclear and cytoplasmic values were then calculated by subtracting their respective average values by the value of the background (a point near each measured cell without fluorescent protein signal). Relative Fluorescence intensity was then calculated by the quotient of the adjusted fluorescence intensity divided by the sum of the adjusted nuclear and cytoplasmic values. Lastly, values were normalized by averaging the first 20 values obtained in the initial 10-minute exposure to find an individualized normalization constant. This constant was subtracted by all of the values for its respective data set.

Protein extraction and Western blot. Pellets of DLD-1 cells were lysed in 2x Laemmli Sample Buffer (Bio-Rad), boiled for 15 min at 98^0C, and ultra-centrifuged at 500 000g (TLA-120.1 rotor) for 10 min at 16^0C. SDS-PAGE and Western blotting were performed as described elsewhere[8]. In brief, the protein samples were separated on 4-20% SDS-PAGE or Bolt™ 8% Bis-Tris gels (Invitrogen) for 1 h and blotted onto PVDF membrane. The membrane was blocked in 5% non-fat milk for 1 h with the following incubation with the primary antibody overnight at 4^0C. The membrane was rinsed and probed for 2 h with the secondary antibodies conjugated to HRP with dilution 1:15000 in 1x TN buffer (150 mM NaCl, 10 mM TrisHCl, pH 7.5, 0.1% Tween 20).

Detection of the signal was performed using FluorChem Imaging System (ProteinSimple) with ECL Prime Western Blotting substrate (GE Healthcare) or SuperSignal WestPico PLUS Chemiluminescent Substrate (ThermoScientific).

1. Holland, A.J., Fachinetti, D., Han, J.S. & Cleveland, D.W. Inducible, reversible system for the rapid and complete degradation of proteins in mammalian cells. *Proc Natl Acad Sci U S A* **109**, E3350-3357 (2012).
2. Morawska, M. & Ulrich, H.D. An expanded tool kit for the auxin-inducible degron system in budding yeast. *Yeast* **30**, 341-351 (2013).
3. Nishimura, K., Fukagawa, T., Takisawa, H., Kakimoto, T. & Kanemaki, M. An auxin-based degron system for the rapid depletion of proteins in nonplant cells. *Nat Methods* **6**, 917-922 (2009).
4. Britton, S., Coates, J. & Jackson, S.P. A new method for high-resolution imaging of Ku foci to decipher mechanisms of DNA double-strand break repair. *J Cell Biol* **202**, 579-595 (2013).
5. Yang, F., Shen, Y., Camp, D.G., 2nd & Smith, R.D. High-pH reversed-phase chromatography with fraction concatenation for 2D proteomic analysis. *Expert Rev Proteomics* **9**, 129-134 (2012).
6. Guo, Y., Bao, Y., Ma, M. & Yang, W. Identification of Key Candidate Genes and Pathways in Colorectal Cancer by Integrated Bioinformatical Analysis. *Int J Mol Sci* **18** (2017).
7. Niopek, D., Wehler, P., Roensch, J., Eils, R. & Di Ventura, B. Optogenetic control of nuclear protein export. *Nat Commun* **7**, 10624 (2016).
8. Blancher, C. & Jones, A. SDS -PAGE and Western Blotting Techniques. *Methods Mol Med* **57**, 145-162 (2001).
