# Supplementary material for: Nucleoporin TPR is an integral component of the TREX-2 mRNA export pathway
Source: Nat Commun. 2020 Sep 11;11:4577. doi: 10.1038/s41467-020-18266-2 (PMC7486939; doi:10.1038/s41467-020-18266-2)
Supplement: Supplementary file 3 — Description of Additional Supplementary Files [file 41467_2020_18266_MOESM3_ESM.pdf]

Description of Additional Supplementary Files

File Name: Supplementary Data 1
Description: Mass-spectrometry data of total cell lysates and NPC-enriched fraction after NUP153, NUP50, TPR, NXF1, or GANP proteins depletion using TMT-assisted and conventional (Labelfree) mass-spectrometry.

File Name: Supplementary Data 2
Description: RNA-Seq data (total RNA, rRNA depletion) after NUP153, NUP50, TPR, RANGAP1, NXF1, or GANP proteins depletion; 3 h of Leptomycin B treatment (inhibition of CRM1 receptor); or 2h of Actinomycin D treatment (transcription block) produced using high throughput sequencing. The supplementary data file contains data for all transcripts (sheet named "All") or transcripts that are 30% significant based on log2FC (sheets named "Protein name_Sig30").

File Name: Supplementary Data 3
Description: Top Gene Ontology (GO) terms (*Biological Process, HumanMine) of differentially expressed transcripts upon loss of NUP153, NUP50, TPR, RANGAP1, NXF1, or GANP. All differentially expressed RNAs are log2FC > 30%.

File Name: Supplementary Data 4
Description: ChIP-Seq data of RNA Pol II Ser5P of AID-TPR cells in the absence (0 h) and presence (2 h) of auxin. Column 2h_0h_Log2FC shows genes with significantly enriched or depleted Ser5P Pol II binding peaks. Column 2h_0h_padj shows adj. p-value.

File Name: Supplementary Data 5
Description: SLAM-Seq data of nascent RNAs labelled with 4-Thiouridine (S4U) in AID-TPR cells in the absence or presence of auxin. Data represented as log2FC of T>C reads counts after 0, 0.5h, 1h, or 2h of S4U incorporation. Log2FC and adj. p-value are shown for the comparison of auxin treated samples to untreated samples with the same labelling time of S4U (for example, t, 2h auxin vs 2h, Figure 5c) or separately for untreated and auxin treated samples where 0.5h, 1h, or 2h S4U labelled samples are compared to 0h labelled samples.
