# Supplementary material for: Nucleoporin TPR is an integral component of the TREX-2 mRNA export pathway
Source: Nat Commun. 2020 Sep 11;11:4577. doi: 10.1038/s41467-020-18266-2 (PMC7486939; doi:10.1038/s41467-020-18266-2)
Supplement: Supplementary file 9 — Reporting Summary [file 41467_2020_18266_MOESM9_ESM.pdf]

nature research

Corresponding author(s): Mary Dasso

Last updated by author(s): Jun 17, 2020

Reporting Summary

Nature Research wishes to improve the reproducibility of the work that we publish. This form provides structure for consistency and transparency in reporting. For further information on Nature Research policies, see our Editorial Policies and the Editorial Policy Checklist.

Statistics

For all statistical analyses, confirm that the following items are present in the figure legend, table legend, main text, or Methods section.

n/a	Confirmed	
☐	☒	The exact sample size (*n*) for each experimental group/condition, given as a discrete number and unit of measurement
☐	☒	A statement on whether measurements were taken from distinct samples or whether the same sample was measured repeatedly
☐	☒	The statistical test(s) used AND whether they are one- or two-sided<br>*Only common tests should be described solely by name; describe more complex techniques in the Methods section.*
☒	☐	A description of all covariates tested
☐	☒	A description of any assumptions or corrections, such as tests of normality and adjustment for multiple comparisons
☐	☒	A full description of the statistical parameters including central tendency (e.g. means) or other basic estimates (e.g. regression coefficient) AND variation (e.g. standard deviation) or associated estimates of uncertainty (e.g. confidence intervals)
☐	☒	For null hypothesis testing, the test statistic (e.g. *F*, *t*, *r*) with confidence intervals, effect sizes, degrees of freedom and *P* value noted<br>*Give P values as exact values whenever suitable.*
☒	☐	For Bayesian analysis, information on the choice of priors and Markov chain Monte Carlo settings
☐	☒	For hierarchical and complex designs, identification of the appropriate level for tests and full reporting of outcomes
☒	☐	Estimates of effect sizes (e.g. Cohen's *d*, Pearson's *r*), indicating how they were calculated

Our web collection on statistics for biologists contains articles on many of the points above.

Software and code

Policy information about availability of computer code

Data collection	No software has been used for data collection.
Data analysis	Ensembl (PMID:27899575), HumanMine database V5.1 2018 (Kalderimis et al., 2014, NAR), UniProt/SwissProt Human database, GRCh38.p7/ hg38, GRCh38.p10. RNAseq mapping: STAR aligner version 2.5.2b (PMID:23104886), Gene expression levels quantification: Counts 1.4.6 (PMID:24227677), differential expression analysis: DESeq2 1.18.1<br>(PMID:25516281), k-means clustering function: R environment 3.3.2, Graphpad Prism version 5.0b, Mascot algorithm (V2.6, Matrix Science Inc.), Venny 2.1 tool, BioVinci software version 1.1.5, r20181005, IGV_2.4.6 software, BWA (VN:0.7.17-r1188), ChIPseq Read quantification: HOMER (PMID: 20513432), differential IP recovery of genes: DESeq2<br>(PMID: 25516281) and MACS2 broad peak calling tools. SLAM-DUNK pipeline, TcReadCounts calculation: DESeq2 (version 1.26.0) using R 3.5.1. Normalization: sizeFactors based on total ReadCounts. Gene patterns analysis: degPatterns from the package DEGreport 1.22.0 with the default options except for minc = 2 and reduce = TRUE. HumanMine database V5.1 2018, V7.0 2020.<br>Microscopy: AxioVision software, AutoQuant software, Volocity, Fiji software version 2.0.0-rc-68/1.52e, Adobe Photoshop. Imaris Surface tool 7.7.2, Percolate algorithm version 3.0.

For manuscripts utilizing custom algorithms or software that are central to the research but not yet described in published literature, software must be made available to editors and reviewers. We strongly encourage code deposition in a community repository (e.g. GitHub). See the Nature Research guidelines for submitting code & software for further information.

Data

Policy information about availability of data

All manuscripts must include a data availability statement. This statement should provide the following information, where applicable:

- Accession codes, unique identifiers, or web links for publicly available datasets
- A list of figures that have associated raw data
- A description of any restrictions on data availability

Data availability. The sequencing data that support the findings of this study have been deposited in NCBI Gene Expression Omnibus (GEO, http://www.ncbi.nlm.nih.gov/geo/) with the accession codes ID GSE132363 (RNA-seq, https://www.ncbi.nlm.nih.gov/geo/query/acc.cgi?acc=GSE132363), GSE148560 (RNA-seq, CRM1, https://www.ncbi.nlm.nih.gov/geo/query/acc.cgi?acc=GSE148560), GSE149890 (SLAM-seq, https://www.ncbi.nlm.nih.gov/geo/query/acc.cgi?acc=GSE149890), and Sequence Read Archive (SRA) under BioProject ID: PRJNA548782 (ChIP Pol II Ser5P, https://www.ncbi.nlm.nih.gov/bioproject/PRJNA548782). The mass spectrometry proteomics data have been deposited to the ProteomeXchange Consortium via the PRIDE67 partner repository with the dataset identifier PXD020075. The data underlying Figures 1c, d, 3b, c, 5b-e, 6b, c, e, f, h, j and Supplementary Figures 2b-d, f-h, j-m, 3b-d, 4c-h, 5d-f, 6a-b, d, f-h, j-l, 7g, h, 8b, 9b, 10 a-n, 11c are provided as a Source Data file. All other data, including immunofluorescence images supporting the finding of this study are available within the article, supplementary figures, and will be provided by the authors upon reasonable request.

Field-specific reporting

Please select the one below that is the best fit for your research. If you are not sure, read the appropriate sections before making your selection.

[x] Life sciences [ ] Behavioural & social sciences [ ] Ecological, evolutionary & environmental sciences

For a reference copy of the document with all sections, see nature.com/documents/nr-reporting-summary-flat.pdf

Life sciences study design

All studies must disclose on these points even when the disclosure is negative.

Sample size	No sample-size calculations were performed. Sample size of n=2-30 was used depending from the variability and consistency of data between groups and assay sensitivity. Detailed information about the number of analyzed fields or cells is provided in the Source Data file. Sample sizes for the experiments were chosen based on literature analysis.
Data exclusions	No data were excluded from the analysis.
Replication	All attempts to reproduce results were successful. RNAseq experiment was triplicated. SLAM-seq experiment was triplicated. ChIPseq experiment was duplicated. Representative images of immunofluorescence and live imaging experiments were performed more than three times. FISH experiments were repeated twice by two independent investigators. For quantification of imaging experiments, we analyzed three independent fields. qRT-PCR data are the result of one representative experiment repeated no less than three times.
Randomization	No samples randomization were performed. This work does not involve participant groups or animals so randomization was not needed for the study.
Blinding	For scoring RNA foci in RNA-FISH experiments, slide identities were blinded. Two independent researches performed RNA metabolic labelling and RNA extraction steps. Identity of samples was blinded before sequencing analysis. At least three unbiased independent investigators performed samples preparation and data analysis for RNAseq, Mass spectrometry, SLAMseq, and ChIPseq analysis. The investigators were not blinded for immunofluorescence, live imaging, and qRT-PCR experiments. Blinding was not relevant for this kind of experiments.

Reporting for specific materials, systems and methods

We require information from authors about some types of materials, experimental systems and methods used in many studies. Here, indicate whether each material, system or method listed is relevant to your study. If you are not sure if a list item applies to your research, read the appropriate section before selecting a response.

Materials & experimental systems

n/a	Involved in the study	
[ ]	[x]	Antibodies
[ ]	[x]	Eukaryotic cell lines
[x]	[ ]	Palaeontology and archaeology
[x]	[ ]	Animals and other organisms
[x]	[ ]	Human research participants
[x]	[ ]	Clinical data
[x]	[ ]	Dual use research of concern

Methods

n/a	Involved in the study	
[ ]	[x]	ChIP-seq
[x]	[ ]	Flow cytometry
[x]	[ ]	MRI-based neuroimaging

Antibodies

Antibodies used

The following antibodies were used:
1. Anti-mouse (A28175, Invitrogen) AlexaFluor-488 conjugated antibodies: https://www.thermofisher.com/antibody/product/Goat-anti-mouse-IgG-H-L-Secondary-Antibody-Oligoclonal/A28175;
2. Anti-rabbit (A11034, Invitrogen) AlexaFluor-488 conjugated antibodies: https://www.thermofisher.com/antibody/product/Goat-anti-Rabbit-IgG-H-L-Highly-Cross-Adsorbed-Secondary-Antibody-Polyclonal/A-11034;
3. Anti-mouse (A11004) AlexaFluor-568 conjugated antibodies (Invitrogen); https://www.thermofisher.com/antibody/product/Goat-anti-Mouse-IgG-H-L-Cross-Adsorbed-Secondary-Antibody-Polyclonal/A-11004;
4. Anti-rabbit (A11011) AlexaFluor-568 conjugated antibodies (Invitrogen): https://www.thermofisher.com/antibody/product/Goat-anti-Rabbit-IgG-H-L-Cross-Adsorbed-Secondary-Antibody-Polyclonal/A-11011;
5. Anti-mouse (A27042) AlexaFluor-680 conjugated antibodies (Invitrogen): https://www.thermofisher.com/antibody/product/Goat-anti-Rabbit-IgG-H-L-Secondary-Antibody-Recombinant-Polyclonal/A27042;
6. Anti-mouse (A-31553) AlexaFluor-405 conjugated antibodies: https://www.thermofisher.com/antibody/product/Goat-anti-Mouse-IgG-H-L-Cross-Adsorbed-Secondary-Antibody-Polyclonal/A-31553;
Specific primary antibodies were used against:
7. Nup50 (A301-782A): https://www.bethyl.com/product/A301-782A/NUP50+Antibody
8. Nup153 (A301-789A): https://www.bethyl.com/product/A301-789A?referrer=search
9. Tpr (A300-828A): https://www.bethyl.com/product/A300-828A?referrer=search
10. GANP (A303-128A) WB: https://www.bethyl.com/product/A303-128A?referrer=search;
A303-127A IF: https://www.bethyl.com/product/A303-127A?referrer=search
11. NXF1 (A303-913A IF: https://www.bethyl.com/product/A303-913A?referrer=search;
A303-915AWB: https://www.bethyl.com/product/A303-915A?referrer=search;
12. Nup133 (A302-386A): https://www.bethyl.com/product/A302-386A?referrer=search;
13. NUP98 (C39A3) Rabbit mAb #2598: https://www.cellsignal.com/products/primary-antibodies/nup98-c39a3-rabbitmab/2598?site-search-type=Products
14. HA (11867423001): https://www.sigmaaldrich.com/catalog/product/roche/roahaha?lang=en&region=US
15. FLAG M2 (F1804): https://www.sigmaaldrich.com/catalog/product/sigma/f1804?lang=en&region=US
16. Actin (13E5, 4970S): https://en.cellsignal.de/products/primary-antibodies/b-actin-13e5-rabbit-mab/4970
17. SON (GTX129778): https://www.genetex.com/Product/Detail/SON-antibody/GTX129778
18. MAB414 (MMS-120P): https://www.biolegend.com/en-us/products/purified-anti-nuclear-pore-complex-proteinsantibody-11498
19. Lamin B2 (33-2100): https://www.thermofisher.com/antibody/product/Lamin-B2-Antibody-clone-E-3-Monoclonal/33-2100
20. Tubulin-alpha (T6199): https://www.sigmaaldrich.com/catalog/product/sigma/t6199?lang=en&region=US
All primary antibodies used for immunofluorescence and WB analysis were diluted 1:200 and 1:1000, respectively. All secondary AlexaFluor-conjugated antibodies were diluted 1:550.

Secondary HRP-conjugated antibodies:
21. anti-mouse: https://www.sigmaaldrich.com/catalog/product/sigma/gena9311ml?lang=en&region=US
22. anti-rabbit: https://www.sigmaaldrich.com/catalog/product/sigma/gena9341ml?lang=en&region=US
23. anti-rat: https://www.sigmaaldrich.com/catalog/product/sigma/a9037?lang=en&region=US
All secondary HRP-conjugated antibodies were diluted 1:10000.

Validation

All the antibodies were already validated and used according to the manufacturer instructions. We listed the antibody registry links to the antibodies used in this study and information regarding validation in the previous box.
The effectiveness of Nup153, Nup50, Tpr, GANP, NXF1, Nup133, and Nup98 antibodies was confirmed after AID-mediated knockdown of the relevant proteins and their subsequent analysis using WB.

Eukaryotic cell lines

Policy information about cell lines

Cell line source(s)

Dukes' type C, colorectal adenocarcinoma (DLD-1), ATCC® CCL-221 cell line was purchased from ATCC. The cell line was subcultured, expanded, and frozen upon receipt. The cells were used within 3-12 passages in all experiments, and were subcultured on DMEM (Life Technologies) supplemented with heat-inactivated 10% FBS (Atlanta Biologicals), antibiotics (100 IU/ml penicillin and 100 ug/ml streptomycin), and 2 mM GlutaMAX (Life Technologies) in 5% CO2 atmosphere at 37°C.
DLD-1 cell line was used for CRISPR/Cas9 modification of endogenous genes. The following cell lines were produced in this study:
AID-NeonGreen-Tpr (reffered as AID-Tpr or AID-NG-Tpr)
AID-HA-Tpr (reffered as AID-Tpr)
AID-NeonGreen-Tpr Nup153-mCherry
AID-NeonGreen-Tpr Nup50-mCherry
AID-Tpr mCherry-NXF1
AID-Tpr mCherry-PCID2
AID-Tpr mCherry-ENY2
Nup153-NeonGreen-AID (reffered as Nup153-AID or Nup153-NG-AID)
Nup153-NeonGreen-AID Nup50-mCherry
Nup153-NeonGreen-AID Tpr-mCherry

	Nup153-NeonGreen-AID mCherry PCID2<br>Nup153-NeonGreen-AID mCherry ENY2<br>Nup50-NeonGreen-AID (reffered as Nup50-AID or Nup50-NG-AID)<br>Nup50-NeonGreen-AID Nup153-mCherry<br>Nup50-NeonGreen-AID Tpr-mCherry<br>Nup50-NeonGreen-AID mCherry PCID2<br>Nup50-NeonGreen-AID mCherry ENY2<br>AID-HA-GANP (reffered as AID-GANP)<br>AID-HA-GANP mCherry PCID2<br>AID-HA-GANP mCherry ENY2<br>AID-FLAG-NXF1 (reffered as AID-NXF1)<br>AID-FLAG-NXF1 mCherry PCID2<br>AID-FLAG-NXF1 mCherry ENY2<br>RanGAP1-NeonGreen-AID (reffered as RanGAP1-AID)
Authentication	The ATCC used SRT and isoenzymes profiles for cell line authentication. All other cell line have been produced based on DLD-1 cell line. Further authentication was not required.
Mycoplasma contamination	DLD-1 and all derived CRISPR/Cas9 clones were tested negative for mycoplasma contamination using custom and published (Young et al., Nature protocols, 2010) primer sets, as well as using Lonza MycoAlert kit, and DAPI staining.
Commonly misidentified lines<br>(See ICLAC register)	No commonly misidentified cell lines were used in the study.

ChIP-seq

Data deposition

☒ Confirm that both raw and final processed data have been deposited in a public database such as GEO.

☐ Confirm that you have deposited or provided access to graph files (e.g. BED files) for the called peaks.

Data access links<br>*May remain private before publication.*	Sequence Read Archive (SRA) under accession ID SUB5824365 (ChIP Pol II Ser5P), BioProject ID: PRJNA548782.
Files in database submission	Input-Tpr-0h-1_S1<br>Input-Tpr-2h-1_S2<br>IP-Tpr-0h-1_S3<br>IP-Tpr-0h-2_S4<br>IP-Tpr-2h-1_S5<br>IP-Tpr-2h-2_S6
Genome browser session<br>(e.g. UCSC)	No Genome Browser session was created.

Methodology

Replicates	Two independent biological replicates were used for library construction using DNA SMART ChIP-Seq Kit.
Sequencing depth	Two hundred million 50 bp paired-end reads were generated for combined replicates, 100 million fragments/pairs.
Antibodies	anti-mouse IgGM-280 (ThermoFisher) dynabeads<br>anti-Ser5P Pol II mouse antibodies (ab5408) from Abcam
Peak calling parameters	Differential IP recovery of genes was tested using DESeq2 (PMID: 25516281) and MACS2 broad peak calling tools.
Data quality	To check ChIP data quality we used MACs. We had 5267 peaks above 5x enrichment and under 5% FDR in the 0hr datasets (untreated Tpr samples), and 7282 in the 2hr datasets (auxin treated Tpr samples).
Software	Alignment of short-reads was performed with BWA (VN:0.7.17-r1188) against reference human genome GRCh38.p7/hg38.<br>Read quantitation over genes was tabulated using HOMER (PMID: 20513432).
